# Effects of Indoor Plants on Human Functions: A Systematic Review with Meta-Analyses

**DOI:** 10.3390/ijerph19127454

**Published:** 2022-06-17

**Authors:** Ke-Tsung Han, Li-Wen Ruan, Li-Shih Liao

**Affiliations:** Department of Landscape Architecture, National Chin-Yi University of Technology, Taichung 411030, Taiwan; ruan@ncut.edu.tw (L.-W.R.); a0401@ncut.edu.tw (L.-S.L.)

**Keywords:** relaxed physiology, diastolic blood pressure, enhanced cognition, academic achievement, volume percentage of the plants, visible greenness rate, dose–response or exposure–outcome relationship

## Abstract

The influences of indoor plants on people have been examined by only three systematic reviews and no meta-analyses. The objective of this study was therefore to investigate the effects of indoor plants on individuals’ physiological, cognitive, health-related, and behavioral functions by conducting a systematic review with meta-analyses to fill the research gap. The eligibility criteria of this study were (1) any type of participants, (2) any type of indoor plants, (3) comparators without any plants or with other elements, (4) any type of objective human function outcomes, (5) any type of study design, and (6) publications in either English or Chinese. Records were extracted from the Web of Science (1990–), Scopus (1970–), WANFANG DATA (1980–), and Taiwan Periodical Literature (1970–). Therefore, at least two databases were searched in English and in Chinese—two of the most common languages in the world. The last search date of all four databases was on 18 February 2021. We used a quality appraisal system to evaluate the included records. A total of 42 records was included for the systematic review, which concluded that indoor plants affect participants’ functions positively, particularly those of relaxed physiology and enhanced cognition. Separate meta-analyses were then conducted for the effects of the absence or presence of indoor plants on human functions. The meta-analyses comprised only 16 records. The evidence synthesis showed that indoor plants can significantly benefit participants’ diastolic blood pressure (−2.526, 95% CI −4.142, −0.909) and academic achievement (0.534, 95% CI 0.167, 0.901), whereas indoor plants also affected participants’ electroencephalography (EEG) α and β waves, attention, and response time, though not significantly. The major limitations of this study were that we did not include the grey literature and used only two or three records for the meta-analysis of each function. In brief, to achieve the healthy city for people’s health and effective functioning, not only are green spaces needed in cities, but also plants are needed in buildings.

## 1. Introduction

Throughout history, humans have valued the health benefits of contact with nature [1]. Theoretically, an evolutionary perspective suggests that evolutionary processes enable humans to respond adaptively and positively to nature [2], whereas a cultural perspective contends that culture affects people’s relations with the natural environment [3]. In line with the evolutionary perspective, the concept of biophilia claims that humans are born with emotional connections with nature and/or other living organisms [4]. This emotional predisposition is deeply embedded in the biological nature of humans and does not disappear even after people leave the natural environment to live a modern urban life [5]. Moreover, the Stress Reduction Theory (SRT; [3]) emphasizes that stress is “the process by which an individual responds psychologically, physiologically, and often with behaviors, to a situation that challenges or threatens well-being” ([6], p. 202), and the natural environment is helpful for recovery from stress, whereas the Attention Restoration Theory (ART; [7]) emphasizes that the natural environment is beneficial to the restoration of directed attention for people’s effective functioning. Empirically, an increasing number of studies on human interaction with nature have demonstrated that contact with nature is favorable to human emotions, physiological functioning, attention restoration, behavior, and health [8,9,10]. Scholars have also conducted systematic reviews (e.g., [11,12,13,14,15] and meta-analyses on related topics (e.g., [16,17,18,19,20,21]). In total, by 2022, more than 60 reviews and meta-analyses regarding nature and health and well-being had been conducted (cf. [22]).

Despite long interests in the theoretical and empirical value of nature to humans, at present, 55% of the world population lives in cities, and the urban population worldwide is expected to increase by 68% by 2050 [23]. For this reason, the World Health Organization Regional Office for Europe in 2016 [24] published a report titled “Urban Green Spaces and Health—A Review of Evidence” to address the importance of nature and green spaces for urban living. The World Health Organization also advocates the healthy city, defined as “one that continually creates and improves its physical and social environments and expands the community resources that enable people to mutually support each other in performing all the functions of life and developing to their maximum potential” [25]. Individuals in contemporary society nevertheless spend most of their time indoors [26], with urban dwellers spending more than 80% of their life indoors [27,28,29]. Moreover, urbanities often do not have ready access to nature [30]. Consequently, urbanites have few opportunities to maintain contact with nature. Although nature includes many elements, plants are the most representative symbol of nature [31,32]. Similarly, “green space” refers to open, underdeveloped, naturally planted land [33], land with grass or trees, or other vegetation region [34]. Studies of indoor nature also tend to focus on plants [35,36]. The exploration of the physical and psychological benefits of indoor plants on people, therefore, merits more attention [37,38]. Interior environments and indoor plants could be important elements of the healthy city. The Rural Development Administration of South Korea suggests placing one small potted plant and one large potted plant per 6 m^2^ floor area in a room to improve the indoor quality [39]. Systematic reviews and/or meta-analyses of the effects of indoor plants on people, however, are far less common than those on natural environments and/or green spaces.

There are only three narrative reviews on the influences of indoor plants related to people. Bringslimark et al. [31] reviewed 21 articles of the experimental research focusing on the benefits of indoor plants on people, which identified benefits such as stress reduction and pain tolerance enhancement. This study was a great stepping stone for later research, particularly with respect to experimental design, measurement, analysis, and reporting. Given that this narrative review was published more than a decade ago, updates are necessary, as is a further distinction of the benefits identified as either self-reported perceptions or objectively measured outcomes using devices or tasks. Deng and Deng [40] reviewed the importance of indoor plants to human health with respect to photosynthesis, transpiration, psychological effects, and air purification, indicating the influence of indoor plants on task performance, health, and stress. Moya et al. [41] reviewed 104 articles published in specific journals between 1984 and 2017 on the influence of vegetation on indoor environmental quality, finding that indoor plants improved people’s comfort, satisfaction, and happiness but presented no strong evidence of improvements in performance and productivity. The inconsistent findings on the influences of indoor plants on participant’s performance [40,41] await further clarification. Further, these three narrative reviews did not follow the rigorous Preferred Reporting Items for Systematic Reviews and Meta-Analyses (PRISMA; [42]), which may have resulted in subjectivity and a lack of transparency and comprehensiveness [43]. They also did not cover studies published in widely used languages, such as Chinese.

Recently, three systematic reviews were conducted to address the gaps left by previous narrative reviews of research on indoor plants and human responses. One followed the Centre for Reviews and Dissemination (CRD) guidelines [44], and two followed the PRISMA guidelines. The first systematic review [45] following the PRISMA covered studies published in English and Chinese—two of the most common languages in the world—focused on self-reported perceptions and included 50 empirical studies, which concluded that the primary beneficial effects of indoor plants were an increase in positive emotions and a reduction in negative feelings, while secondary benefits included a reduction in physical discomfort. The second systematic review [36] following the CRD covered 26 studies of the health and well-being impacts of indoor nature (actual and simulated plants and aquariums) on the elderly and concluded that higher-quality studies showed that indoor gardening programs were helpful for cognition, psychological well-being, social outcomes, and life satisfaction. The third review [35] following the PRISMA covered 37 studies published in English and Dutch on the influences of indoor and outdoor nature on adolescents, which found associations between outdoor campus green space and enhanced quality of life and perceived restoration. The common findings of the two systematic reviews are that indoor plants benefit psychological well-being [36,45]. Self-reported psychological responses, however, may be different from actual human functions (cf. [46]). Moreover, Yeo et al. [36] researched only older adults and did not specifically focus on indoor plants. Van den Bogerd et al. [35] researched only adolescents and did not specifically focus on indoor plants. Furthermore, none of these three reviews conducted meta-analyses to provide quantitatively synthesized evidence of the effects of indoor plants on humans. This may be because of the heterogeneity of the outcomes.

Given the above-mentioned factors, the purpose of the present study was to perform a systematic review with meta-analyses of Chinese and English empirical quantitative research on the influences of indoor plants on human functions, in order to address the current research gap of the lack of meta-analyses on this subject and to respond to the fulfillment of the daily functions of urbanites as advocated by the promotion of the healthy city. Specifically, the objective of this study was to examine if the presence of indoor plants of any type serving as an intervention has any objectively measured effects, such as using devices, tasks, examinations, or performance records, on human functions. We reviewed all research with any study design that assessed all human functions exposed to indoor plants against those exposed to no indoor plants or other elements. Accordingly, the review question was whether indoor plants have any effects on human functions. The present systematic review may provide more comprehensive information associated with the aforementioned effects and serve as a reference for future research. This study identifies what empirical and quantitative studies of human functions have been performed in relation to indoor plants, particularly regarding research validity (cf. [31]), such as plant quantity measurements (construct validity: number, size, volume percentage, and green coverage ratio), potential effect modifiers (confounder: exposure duration, distance to plants, room climate, and room size), funding (conflict of interest), and what and how further research could be performed. The meta-analyses further examined the overall results of studies exploring the effects of indoor plants on human functions, rather than reviewing studies individually. Systemic reviews and meta-analyses are effective in providing optimal synthesis evidence, and the constantly updated data may serve as a basis for policy making [43], such as regarding the healthy city or effective human functions [25]. The systematic review and meta-analyses conducted in this study are the first to provide a synthesis of the quantitative evidence regarding the specific effects of indoor plants on human functions.

## 2. Methods

This study followed the PRISMA guidelines, particularly their specific checklist items and item orders, although PRISMA focuses on evaluating interventions in the field of medical care [42]. The conduct of the review involved no significant deviations from the protocol, except that we searched two more databases than the protocol.

### 2.1. Eligibility Criteria

The eligibility criteria for inclusion of a study in this research were as follows: (1) participants of any type were recruited; (2) no criteria were set for the type of indoor plant to be used in interventions; (3) the comparator was participants in an indoor environment without any plants or with other elements; (4) the outcome included any type of objectively measured human function, such as use of devices, performance tasks, examinations, or records, rather than self-reports; (5) all types of study design were included; and (6) the language was either English or Chinese. Because Chinese and English are the most commonly used languages in the world, studies written in these two languages were selected to decrease the risk of language bias [43]. Other languages were not included because of limited resources.

### 2.2. Information Sources

The information sources included four electronic databases, of which the Core Collections hosted by Web of Science (1988–) and Scopus (1970–) are English-language databases, while the Journal Collections hosted by WANFANG DATA (1980–) and Taiwan Periodical Literature (1970–) are Chinese-language databases. At least two databases, therefore, were searched for each of the two languages. The final search on WANFANG DATA was performed on 14 August 2019, while that on the Web of Science was performed on 11 November 2019. The search on Taiwan Periodical Literature was performed on 21 October 2020, and the search on Scopus was performed on 13 November 2020. We searched WANFANG DATA and Web of Science in the first round and Taiwan Periodical Literature and Scopus in the second. About one year thus elapsed between the searches of the two rounds. The follow-up searches of all four databases were completed on 18 February 2021. The coverage cutoff date was 31 December 2020. We decided that the coverage ended at the end of 2020 rather than in the middle of the year, so later updated searches could continue at the start of 2021. Moreover, two supplementary approaches to identifying studies were applied: one was that the related studies were identified by reference searches of the included studies, while The other was contact with the authors of the included studies to seek missing information, particularly regarding the results.

### 2.3. Search

The search terms included the following: “indoor”, “interior”, “architecture”, “building”, “plant”, “vegetation”, “greening”, “greenery”, “green”, “greenness”, “perception”, “psychology”, “emotion”, “physiology”, “cognition”, “restoration”, “behavior”, “health”, and “performance”, as found in previous studies [8,45,47] and in peer reviewers’ suggestions. In the Boolean search, only “AND” was adopted as the operator, as in (1) indoor “AND” plant “AND” perception, or (2) architecture “AND” greening “AND” psychology. Except for the eligibility criteria and the search coverage, we did not have any restrictions such as topics, keywords, or dates. The full search strings applicable to all four databases are listed in Supplementary Material Table S1.

### 2.4. Study Selection

The present systematic review included only quantitative empirical studies published in journals, primarily because of their relatively easy accessibility. Technical reports, proceedings, books, and unpublished theses or dissertations (i.e., grey literature) therefore were not considered. Empirical studies using plants in a room or building as the intervention, irrespective of how many plants, what sizes, what types, foliage or floral, actual or virtual, duration of presence, or distance from the participants, were included in accordance with the eligibility criteria. “Empirical research” refers to the analysis of real data, and quantitative research uses computation, mathematics, and statistics to explore the target phenomenon [48]. Regarding the causal relationship between variables in quantitative research, randomized controlled experiments, in which participants are randomly assigned to experimental and control groups (also referred to as “RCT” in clinical professions), provide higher-quality results than nonrandomized controlled quasi-experiments, in which participants are not randomly assigned to experimental and control groups (also referred to as “non-RCT”), and quasi-experiments outperform surveys [49]. Field experiments conducted in real-world environments, however, exhibit more favorable ecological validity than laboratory experiments [50]. If surveys were used to collect objective outcomes such as health indicators, they were also included.

### 2.5. Data Collection Process

L.-W.R. performed searches with the abovementioned terms in the databases and reviewed study titles and abstracts that met the eligibility criteria. L.-S.L. independently performed searches with the same terms in the same databases and reviewed study titles and abstracts that met the eligibility criteria. As a result, L.-W.R. and L.-S.L. had an agreement rate of 99.9% on both Web of Science and WANFANG DATA, respectively, and L.-S.L. and K.-T.H. also had an agreement rate of 99.9% on Taiwan Periodical Literature. In cases where the title and abstract were insufficient to determine the study’s eligibility, L.-W.R. or L.-S.L. proceeded to read the full text. All the studies of each included paper were reviewed. Then, K.-T.H. reviewed the extracted full-text studies that met the eligibility criteria. K.-T.H. and L.-W.R. conducted data extraction and quality appraisal. Initial disagreement regarding a study’s opinion was resolved by discussion between the two reviewers.

### 2.6. Data Items

The following 14 data items were extracted from the reviewed records: sources, participants, interventions, comparator, exposure duration, distance to plants, room climate, room size, study design, functions, function categories, outcomes, funding, and languages.

### 2.7. Risk of Bias in Individual Studies

The included studies were analyzed in accordance with the quality appraisal system proposed by Ohly et al. [19]. This appraisal system comprises 19 appraisal items, including quality indicators from the CRD [44], critical appraisal checklists from the Critical Appraisal Skills Program [51], and quality assessment tool for quantitative studies from the Effective Public Health Practice Project [52]. We adopted the quality appraisal system because it was more comprehensive and current than other appraisal systems. This appraisal system, which has an option of criterion inapplicable to this study design, was applied to both RCTs and nonrandomized studies [19,20].

### 2.8. Summary Measures

The summary measures of this study included data measured in empirical research using devices, tasks, academic achievement scoring, and actual health indicators. These data were reviewed to examine the influences of indoor plants on participants’ functions.

### 2.9. Planned Methods of Analysis

Where sufficient studies (at least two studies) using comparable outcome measures allowed us to conduct meta-analyses, the present meta-analyses reported the means and standard deviations (SDs) of each function category. Comprehensive Meta-Analysis version 3 (Biostat, Englewood, NJ, USA) was applied to conduct Cochran’s Q tests and draw forest plots as well as to analyze pooled effect sizes, sensitivities, and publication biases. Because the measurement of functions in similar categories varied between the records, the measurement outcomes of these categories were processed using the standardized mean difference (SMD), thereby providing an indicator enabling the comparison and synthesis of function outcomes in these categories (cf. [43]). For records using the same methods to measure function in the same categories, outcomes were, in general, directly compared and synthesized to determine the mean differences (MDs). For studies with more than one experimental group [53], each experimental group and control group was separately analyzed [43]. Specifically, Cochran’s Q test was performed to examine whether the classification outcomes of function in each category were heterogeneous or homogenous. Additionally, forest plots were adopted to present the relative importance and research outcome directions among the studies visually. Subsequently, the pooled effect size was computed using fixed-effect or random-effect models.

### 2.10. Risk of Bias across Studies

The meta-analyses used funnel plots and Egger’s regressions to identify potential publication bias on the basis of the research results of each function category.

### 2.11. Additional Analyses

The meta-analyses included the sensitivity analysis of the results of the records for each function category, which examined if the pooled effect sizes changed notably when any of the records was removed—an indication of the stability of the results.

## 3. Results

### 3.1. Study Selection

**Figure 1:**
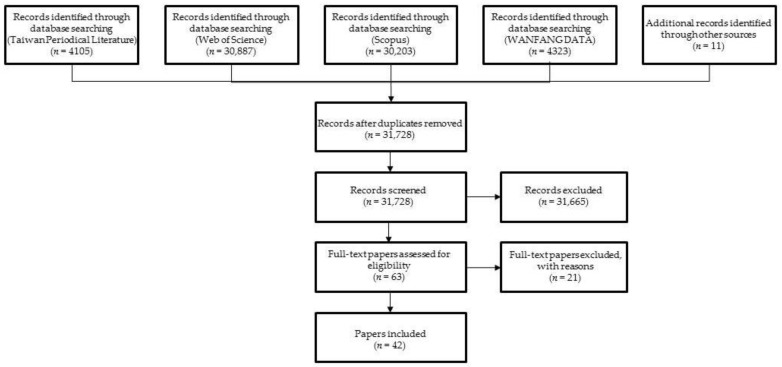
Flow chart of the screening process.

The abovementioned terms were used to search the four databases separately. The search yielded 30,887 records from the Web of Science, 4323 from WANFANG DATA, 30,203 from Scopus, and 4105 from Taiwan Periodical Literature. Repeated records were excluded. Moreover, 11 records were identified after searching the references of the searched papers (which were considered as other sources), resulting in 31,728 journal articles in total. Papers with titles and abstracts meeting the eligibility criteria were identified, resulting in 63 preliminarily qualified papers. The full texts of these 63 papers were extracted for further scrutiny, and 21 studies that failed to meet the criteria were excluded. As a result, 42 qualifying records were included (Figure 1). The major reasons for excluding records were that the research was not empirical and quantitative, human functions were not objectively measured, and plant functions were measured (Supplementary Material Table S2).

### 3.2. Study Characteristics

Among the 42 journal articles included in the systematic review, 5 (11.9%) and 37 (88.1%) were written in Chinese and English, respectively. The earliest paper was published in 1996, and the latest in 2020 (the terminus of the search coverage). The 25 year coverage period was divided into 5 year intervals to analyze the number of publications during each interval. The number of published papers increased relatively steadily (Table 1).

In terms of geographical distribution, most of the studies were from China (10; 23.8%), followed by the United States (8; 19.0%), Japan (6; 14.3%), South Korea (5; 11.9%), and Taiwan (4; 9.5%). Asia was thus the leading continent, followed by America, Europe, and Africa. Most studies were from the Global North, followed by the Global South and the Equatorial region (Table 2). Detailed statistics could not be compiled because not every record provided the participants’ socioeconomic backgrounds. The majority of the participants, however, were college students. Only six studies recruited office workers [54,55,56,57,58,59], five studies recruited patients [60,61,62,63,64], two studies recruited junior high school students [65,66], and one study recruited high school students as participants [67] (Table 2).

The records generally did not focus on only one measure of human functions. The reviewers found that they examined 52 functions. Most of the records were related to physiology (27; 51.9%), followed by cognition (15; 28.8%), health, (seven; 13.5%), and behavior (three; 5.8%). Concerning study design, most of the records adopted experimental methods (26; 61.9%). These were followed by those conducting field experiments (seven; 16.7%), field quasi-experiments (five; 11.9%), and surveys (four; 9.5%). Among the 42 records, 20 reported the specific number of indoor potted plants as the intervention; the highest number of potted plants was 34 [66], and the lowest number of potted plants was one [56,59,60,68,69,70,71]. Three papers reported the green coverage ratio, with the highest at 10% and the lowest at 3% [57]. Two papers also indicated the volume of indoor plants as a percentage of the total experimental space, where the largest volume was 17.9% [53] and the smallest was 5% [67] (Table 3). In addition, three records used photographs or slides as surrogates for indoor plants [72,73,74], and one paper employed virtual-reality plants [75] (Table 4).

A total of 34 papers recorded the time during which participants were exposed to indoor plants. Among these, the longest exposure time was one year [76] and the shortest was 15 s [72]. Thirty-three papers reported the room size. The experiment room used by Toyoda et al. [59] was the largest in terms of its floor area (1260 m^2^), and that used by Genjo et al. [57] was the largest in terms of its volume (675 m^3^). By contrast, the room used by Kim et al. [77] was the smallest in both floor area (7.26 m^2^) and volume (14.52 m^3^). Among the records, only 13 reported the participant–plant distance in a room, with the greatest distance being 3 m [72] and the smallest being 0.38 m [60] (Table 3).

Some records also provided data on the ambient environment in which the plants were placed. Specifically, 19 papers recorded the room temperature, with the highest being 27 °C [78] and the lowest 20 °C [59]. Humidity was reported in 13 papers, with the highest value at 70% [79] and the lowest 34% [80]. Only one record measured wind speed (0.2 m·s^−1^; [81]). Twelve records indicated lighting, of which only one adopted the quantum as the lighting unit (10.6 μmol m^−2^·s^−1^; [69]), whereas the remaining 11 used illuminance as the unit. The most intense lighting was 1365.5 lux [82], while the least intense lighting was 300 lux [56] (Table 3).

Among the 42 records, only 18 indicated their funding sources. Most of the funding sources were in governmental sectors, while only two may be from stakeholders ([83], American Horticultural Therapy Association; [68], The Swedish Flower Corporations) (Table 4). Funding from stakeholders might cause a conflict of interest.

**Table 4:** Summary of the study characteristics of the records.

Source	Participant	Interventions	Comparator	Exposure Duration	Distance to Plants	Room Size	Room Climate	Study Design	Functions	Function Category	Funding	Publication Language
[78]	96 US adults (48 males and 48 females, 80 of whom were college students), age: 18 to 46	Presence or absence of 17 potted plants in a computer lab	Control			13.5 × 7.3 × 2.6 m	27 °C, 38% RH, 420 lux	Field experiment (RCT)	SBP, reaction time	Physiology, cognition		English
[53]	81 US adults	10 potted plants (accounting 7.16% of the space), 22 potted plants (accounting 17.88% of the space), or no plants in an office	Control	15–20 min		12.08 m^2^, 31.3 m^3^		Field experiment (RCT)	A sorting task, a productivity task	Cognition		English
[84]	814 Chinese participants (347 males and 467 females), ethnicity: Asian	A building with or without indoor greening						Survey (non-RCT)	Neurobehavioral Functioning Evaluation System Testing	Cognition	Sciences and Technology Commission of Shanghai	Chinese
[80]	198 US adults (71 males and 127 females), 176 of whom were college students	5 potted plants, nonplant objects, no plants in a room	Nonplant objects, control	about 17 min		3.5 × 6 × 2.4 m	23 °C, 34% RH, 703 lux	Experiment (RCT)	Skin temperature, blood pressure, pain tolerance	Physiology, behavior		English
[83]	150 US college students (75 males and 75 females), mean age: 19.6	9 potted red-flowering geraniums, 9 potted non-flowering geraniums, no plants in a lab	Non-flowering plants, control	5 min	1.8 m		22.4 °C	Experiment (RCT)	EEG, EDA, finger skin temperature	Physiology	American Horticultural Therapy Association	English
[70]	146 Japanese college students (83 males and 63 females), ethnicity: Asian	1 potted 1-m-tall plant placed in front of the participant, the same plant placed on the right-hand side of the participant, no plants in a room	Control	15 min	2.345 m in front of and 1.75 m at the side of the participants	5.81 × 2.78 × 2.35 m		Experiment (RCT)	An association task, a sorting task	Cognition		English
[69]	66 US college students (32 males and 34 females), age: 91% from 18 to 24	1 potted flower arrangement (45 × 45 × 45 cm), lavender fragrance, flower and fragrance, or no plants and no fragrance in a lab	Control	30 min		3.5 × 2.7 × 2.4 m	21 °C, 10.6 μmol·m^−2^·s^−1^	Experiment (RCT)	EEG, EDA, skin temperature	Physiology		English
[85]	90 US college female students, mean age: 18.9	Foliage and flowing plants, flowing plants, or no plants in a lab	Control	5 min maximum	1.4 m	3.9 × 2.3 × 2.7 m	21.7 °C, 904 lux	Experiment (RCT)	Pain tolerance, EEG, EDA, finger skin temperature	Behavior, physiology		English
[71]	90 Japanese college students (35 males and 55 females), ethnicity: Asian	1 potted 1.5-m-tall plant, a magazine rack put at the same location, or no plants and no magazine racks in a room	A magazine rack, control	15 min	About 2.9 m in front of the participant	2.78 × 5.81 × 2.35 m		Experiment (RCT)	An association task	Cognition		English
[72]	38 Taiwanese college students (10 males and 28 females), ethnicity: Asian	Presentation of 6 slides (office without a window view nor indoor plants, office without a window view but with indoor plants, office with a city window view but without indoor plants, office with a city window view and with indoor plants, office with a nature window view but without indoor plants, and office with a nature window view and with indoor plants) in a lab	Control	15 s for each slide	3 m	7 × 5 m	25 °C	Experiment (non-RCT)	EEG, EMG, BVP	Physiology		English
[55]	364 Norwegian office workers, mean age: 43.1	Presence or absence of potted plants on desks or shelves in an office						Survey (non-RCT)	Sick leave	Health		English
[68]	50 healthy Swedish people (23 males and 27 females), mean age: 39.2	1 potted flowering begonias (*Begonia Elatior*) approximately 22 cm high (control plant irrigated with ordinary local tap water; experiment plant irrigated with vortex-rotated local tap water) in an office	Plant irrigated with ordinary local tap water	10 min for each plant		5.6 × 3.0 × 2.4 m	23–24 °C, 36–38% RH, 570–650 lux	Experiment (RCT)	Heart rate, heart rate variability, power spectral density	Physiology	The Swedish Flower Corporations	English
[63]	90 South Korean patients who had received appendectomy (52 males and 38 females), mean age: 37.6, ethnicity: Asian	Presence or absence of 12 potted flowering plants in a ward	Control					Field experiment (RCT)	Pain killer consumption, blood pressure, body temperature, heart rate, respiratory rate	Health, physiology		English
[67]	140 South Korean female high school students, ethnicity: Asian	Presence or absence of plants in 2 classrooms (accounting for 5% of the space)	Control	14 weeks of school time				Field quasi-experiment (non-RCT, pre-post design)	Cortisol level, health	Physiology, health		English
[86]	89 US sophomores	Presence or absence of plants in a classroom	Control	1 semester of class time				Field quasi-experiment (non-RCT)	Course grade	Cognition		English
[65]	76 Taiwanese junior high school students (58 males and 18 females), mean age: 13.55, ethnicity: Asian	Presence or absence of 6 potted plants (about 135 × 80 cm, having a green coverage ratio of 6%) in a classroom	Control	12 weeks of school time				Field quasi-experiment (non-RCT)	Sick leave, misconduct	Health, behavior		English
[64]	80 South Korean female patients who had received thyroidectomy, mean age: 36.2, ethnicity: Asian	Presence or absence of 12 potted flowering plants in a ward	Control					Field experiment (RCT)	Pain killer consumption, hospitalization days	Health		English
[87]	34 Norwegian college students (12 males and 22 females), mean age: 24.15	Presence or absence of 4 potted plants (2 flowering pink *Phalaenopsis*, 1 30-cm-tall *Aglaonema commutatum*, and 1 120-cm-tall *Schefflera arboricola*) in an office	Control	60 min		3.9 × 2.1 × 3.6 m		Experiment (RCT)	The Reading Span Task	Cognition		English
[66]	36 Taiwanese junior high school students (18 males and 18 females), mean age: 12.41, ethnicity: Asian	Taking care of 34 potted plants inside and outside a classroom (with a green coverage ratio of 6.3% indoors)	Control	18 weeks of school time				Field experiment (RCT)	Examination score	Cognition		Chinese
[73]	30 Chinese college students (15 males and 15 females), ethnicity: Asian	Presentation of 5 photos of vegetation landscapes and a blank in a room	Control	2 min	0.5 m	7 × 4 × 3 m	25 °C, 40% RH	Experiment (RCT)	ECG, blood pressure, heart rate, GSR, fingertip pulse	Physiology		English
[74]	30 Chinese college students (15 males and 15 females), age: 18 to 24, ethnicity: Asian	Presentation of 12 photos of flowers and a blank in a room	Control	2 min	0.5 m	7 × 4 × 3 m	25 °C, 40% RH	Experiment (RCT)	Blood pressure, heart rate, GSR, fingertip plus	Physiology	National Key Technology Research and Development Program in China	English
[88]	29 Japanese college students (14 males and 15 females), age: 19 to 24, ethnicity: Asian	Potted *Hedera helix* L. (60 × 40 cm) of 5 different colors on a table in a room	Different colors of the plant	1 min for each plant color	0.5 m			Experiment (RCT)	Brain activity, eye movement	Cognition	Egyptian Ministry of Higher Education	English
[89]	28 Japanese undergraduate and graduate students (14 males and 14 females), mean age: 21.42, ethnicity: Asian	Placement of 1 potted plant of 3 different colors on a table in a room	Different colors of plants	1 min for each plant color	1.5 m	59.4 m^2^	23 °C, 55% RH, 700 lux	Experiment (RCT)	Eye movement, brain activity	Cognition	Egyptian Ministry of Higher Education	English
[79]	30 South Korean college students (15 males and 15 females), mean age: 23.5, ethnicity: Asian	Placement of potted plants (60 × 40 cm) of 5 different colors on a box in a classroom	Different colors of plants	3 min for each plant color	1 m	7 × 4.5 × 2.8 m	25 °C, 70% RH, 700 lux	Experiment (RCT)	EEG	Physiology		English
[58]	Study 3: 33 British adult office workers (16 males and 17 females), mean age: 28	Study 3: presence or absence of 8 potted plants (average height 90 cm) in an office	Control					Study 3: Field experiment (RCT)	An information management and processing task, a vigilance task	Cognition		English
[81]	16 Chinese college students (8 males and 8 females), mean age: 23.5, ethnicity: Asian	Presence of potted plants of the combinations of 3 colors, 3 scents, and 3 sizes on a table in an office	Combinations of plant colors, scents, and sizes	10–15 min			22 °C, 41.65% RH, 0.2 ms^−1^ wind velocity	Experiment (RCT)	EEG, ECG, oxyhaemoglobin saturation, fingertip blood flow, skin resistance, respiration rate	Physiology	Sciences and Technology Commission of Shanghai	English
[82]	24 South Korean male adults, mean age: 24.9, ethnicity: Asian	A plant transplanting task, a computer operation task on a table in a greenhouse room	A computer task	15 min			20.8 °C, 57.7% RH, 1365.5 lux	Experiment (RCT)	Heart rate variability, blood pressure, pulse rate	Physiology		English
[54]	565 Norwegian office workers	Outdoor nature contact, indoor nature contact, and outdoor view through windows						Survey (non-RCT)	Sick leave	Health		English
[61]	270 Pakistani surgical patients, ethnicity: Asian	Presence or absence of foliage plants and flower arrangements in a ward	Control					Field experiment (RCT)	Blood pressure, heart rate, respirationrate, body temperature, hospitalization days, analgesics consumption	Physiology, health	The University of Agriculture Peshawar in Pakistan	English
[90]	30 Egyptian male college students, age: 22 to 37, ethnicity: African	Potted *Hedera helix* L. (60 × 40 cm) of 5 different colors on a table in a room	Different colors of the plant	1 min for each plant color	0.5 m	59.4 m^2^	21 °C, 55% RH	Experiment (RCT)	Eye movements, brain activity	Cognition, physiology	Egyptian Ministry of Higher Education	English
[91]	5 Indonesians, ethnicity: Asian	A room with 5 potted plants and a room without plants	Control	30 min				Experiment (non-RCT)	Heart rate, blood pressure	Physiology	Ministry of National Education in Indonesia	English
[77]	66 Hong Kongese college students (40 males and 26 females), mean age: 25.6, ethnicity: Asian	A basement room with plants, with a fake window, with plants and a fake window, and without plants nor a window	Control	At least 8 min		3.3 × 2.2 × 2 m	24 °C	Experiment (non-RCT)	EDA, a response time task	Physiology, cognition	Hong Kong Polytechnic University	English
[75]	28 US adults (12 males and 16 females), age: 23 to 42	Presence or absence of plants in an actual environment and a virtual one	Control	5 min				Experiment (RCT)	Heart rate, EDA, blood pressure, a visual reaction time task, The Stroop task, a visual backward digit span task	Physiology, cognition	Campus Sustainability Innovation Fund, Harvard University Office for Sustainability	English
[57]	36–41 Japanese office workers, mean age: 33.95, ethnicity: Asian	Presence (3–10% green coverage ratio) or absence of plants in 2 offices	Control	16 weeks of working hours		132 m^2^ (321 m^3^), 270 m^2^ (675 m^3^)		Field quasi-experiment (non-RCT)	Heart rate, salivary amylase activity, critical flicker fusion frequency, fingertip pulse wave	Physiology	Grant-in-Aid for Scientific Research, Japan Society for the Promotion of Science	English
[60]	50 Chinese female elders with hypertension, mean age: 79.2, ethnicity: Asian	Presence or absence of 1 potted plant on a table in a room	Control	5 min	0.38 m		23 °C, 40% RH, 500 lux,	Experiment (RCT)	Blood pressure, EEG	Physiology		English
[76]	100 Taiwanese elders, age: >65, ethnicity: Asian	Presence or absence of plants in houses		1 year				Survey (non-RCT)	Blood pressure, heart rate	Physiology	Ministry of Science and Technology in Taiwan	English
[59]	63 adult Japanese office workers (33 males and 30 females), mean age: 40.15, ethnicity: Asian	Presence or absence of 1 potted plant (15–20 cm tall, 7–10 cm wide) on the desk in an office	Control	3 min		1260 m^2^	20–24 °C, 40–50% RH, 500–700 lux	Field experiment (non-RCT, pre-post design)	Pulse rate	Physiology		English
[56]	30 Chinese female office workers, mean age: 29.42, ethnicity: Asian	Presence or absence of 1 potted plant with blue or purple flowers on a desk in an office	Control	3 min	0.4 m		21 °C, 50% RH, 300 lux	Field quasi-experiment (non-RCT, pre-post design)	EEG, heart rate variability, skin conductance	Physiology	National Nature Science Foundation of China	English
[92]	33 Chinese elders, age: 65 to 99, ethnicity: Asian	Combination of potted succulents (3–10 cm tall, 3 cm wide) or flower arrangement (50–60 cm tall, 5–18 cm wide) performed indoors	Flower arrangement	25 min				Experiment (RCT)	Salivary cortisol	Physiology	National Nature Science Foundation of China	Chinese
[62]	34 Chinese elders with dementia (13 males and 21 females), ethnicity: Asian	With or without a treatment course of indoor horticultural activities (sowing, transplanting seedlings, succulents potting, and herbal flower potting)	Control	30 min				Experiment (non-RCT)	Blood pressure, heart rate, ECG	Physiology	National Nature Science Foundation of China, Beijing Science and Technology Project Foundation	Chinese
[93]	44 Chinese elders living alone, ethnicity: Asian	Four kinds of indoor horticultural activities (sowing, transplanting seedlings, succulents potting, and herbal flower potting)	Within- participants, between-participants	30 min				Experiment (non-RCT)	Blood pressure, heart rate, ECG	Physiology	Beijing Science and Technology Commission Green Communication Foundation	Chinese
[94]	Study 1: 120 South Africans, mean age: 33.72, ethnicity: African	Presence of 3 potted plants, 6 plant pictures on 3 walls (80 × 80 cm), and no potted plants and plant pictures in an office	Control	35 min		3 × 3 m	21 °C, 510 lux	Experiment (RCT)	A card-sorting task, a reading task	Cognition		English

RH: relative humidity; SBP: systolic blood pressure; DBP: diastolic blood pressure; EEG: electroencephalography; EDA: electrodermal activity; EMG: electromyography; BVP: blood volume pulse; ECG: electrocardiography; GSR: galvanic skin response; RCT: randomized controlled trial; non-RCT: not randomized controlled trial.

### 3.3. Risk of Bias within Studies

Most of the included studies (90.5%) applied quasi-experimental or experimental methods. Control and experimental groups were therefore involved. In quasi-experimental research, particularly field research (11.9%), researchers were unable to assign interventions randomly to participants as is the case in clinical trials. Surveys, field quasi-experiments, and quasi-experiments, therefore, could not achieve sequence generation, which reduces the risk of bias. In addition, concealing the intervention assignment from participants was difficult because indoor plants were easily noticed in a room, resulting in lower allocation concealment ability. Similarly, blinding participants concerning their intervention was also challenging. Furthermore, the risk of incomplete data on outcomes caused by participant attrition and exclusion might exist because the included studies seldom mentioned participant attrition or exclusion.

The mean quality appraisal score of the 42 records was 17.2 points out of a possible 38, i.e., 45.3% (17.2/38 = 45.3%) of the total, indicating moderate research quality (high: 67–100%, moderate: 34–66%, low: 0–33%; [19]). The five items (of a total of 19) in the quality appraisal system for which the records included scored lowest are discussed next. First, none of the 42 papers complied with the intention-to-treat (ITT) analysis (0%) (i.e., all data were included after allocation). Additionally, the participants were not sufficiently representative because most were students (17 studies included college students, 1 study included high school students, and 2 studies included junior high school students). Only 5 papers involved general adult participants, whereas the remaining papers involved patients or office workers. The second lowest score regarding the quality appraisal system was found in the only 1 paper (2%) in which the outcome assessors were completely unaware of participant allocation. The third lowest scores were observed in the following two items of the quality appraisal system: only 2 papers (5%) reported statistical power and randomization procedure, respectively (Table 5 and Table 6 respectively). The records exhibited desirable quality in the following items of the appraisal system: (1) all the papers (100%) included individual level analyses, (2) data collection in 39 studies (95%) was consistent, (3) a total of 37 studies (90%) provided a clear description of interventions and control, and (4) 32 papers (78%) accounted for all participants and applied statistical analysis methods appropriate for study design.

### 3.4. Results of Individual Studies

The research outcomes of each study for the systematic review are summarized in Table 7. In brief, the systematic review concluded that indoor plants, in general, affect participants’ functions positively, particularly their physiology and cognition. Regarding physiological functions, participants exhibited greater benefits in a room with plants than in a room without plants in relation to lower blood pressure [60,61,63,76,78,82], lower electrodermal activity (EDA) [69,83,85], lower electroencephalography (EEG) α and β waves [56,69,72,81,83], lower heart rate [59,61,62,63,68,76,91,93], and lower respiration rate and body temperature [61].

Regarding cognitive functions, when indoor plants were present, participants exhibited higher academic achievement [66,86] and better performance in various cognitive tasks [58,71,75,77,78,84,87,94]. In health-related functions, with exposure to indoor plants, participants less frequently took sick leave [54,55,65,67], consumed fewer pain killers [61,63,64], and had fewer hospitalization days [64] than participants in environments where indoor plants were absent. In behavioral functions, participants presented greater pain tolerance of putting hands in cold water [80,85] and less misconduct [65] when indoor plants were in the room than when indoor plants were not in the room.

### 3.5. Synthesis of Results

The data for the meta-analyses included only the participants’ physiological functions (i.e., diastolic blood pressure (DBP), EEG α and β waves) and cognitive functions (i.e., attention, academic achievement, and response time) because at least two studies are needed to conduct the meta-analyses. Given that the number of the records of each of the function categories was small, randomized control trials and non-randomized studies of interventions were included for the meta-analyses. Moreover, various interventions of indoor plants regardless of species, type, quantity, exposure time, and distance to participants were dichotomized as groups with plants and groups without plants.

### 3.6. DBP

Three papers examining the influence of indoor plants on DBP, which was measured by sphygmomanometers measured in mmHg, were included for the meta-analysis (Table 8). In total, 248 participants were evenly exposed to conditions either with plants or without plants. Lee et al. [82] recruited only male adults in South Korea, whereas Hassan et al. [60] recruited only female older adults with high blood pressure in China. Chen et al. [76] surveyed male and female elders in Taiwan six times over one year. Both Lee et al. [82] and Hassan et al. [60] randomly assigned their participants to different groups, while Chen et al. [76] did not. All three papers were appraised as having moderate research quality.

**Figure 2:**
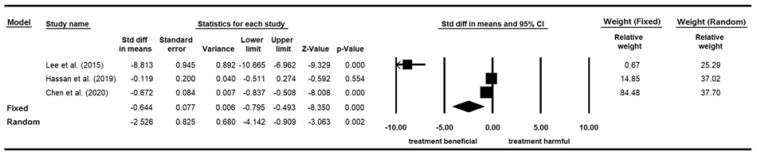
Forest plot of studies on the influence of indoor plants on DBP [60,76,82].

The heterogeneity test of the three studies focusing on DBP revealed a significant difference (*p* < 0.05) with I^2^ = 97.554%, confirming high heterogeneity among the studies. A random-effect model was therefore applied. Given that the standard deviation (SD) of one study was much smaller than that of the other two, SMD, rather than MD, was adopted here. The pooled effect size (SMD) was −2.526 with a 95% confidence interval ranging between −4.142 and −0.909. The results indicated that the group with plants had significantly (*p* = 0.002) lower DBP values than the group without plants (Table 9). The relative weight of both the Hassan et al. [60] and Chen et al. [76] studies was about 37.00%, and that of Lee et al. [82] was 25.29% (Figure 2).

### 3.7. EEG α Waves

Three papers examining the influence of indoor plants on EEG α waves, which was measured by brain activity instruments with Hertz as the unit of measurement, were included for the meta-analysis (Table 10). The studies had a total of 200 participants. Among them, 85 were in the control group (without plants) and 115 in the experimental group (with plants). Chang and Chen [72] recruited college students in Taiwan and Qin et al. [81] recruited college students in China, whereas Elasdek and Liu [56] recruited only female office workers in China. Chang and Chen [72] and Elasdek and Liu [56] did not randomly assign their participants to different groups, while Qin et al. [81] did. These three papers were appraised as having moderate research quality.

**Figure 3:**
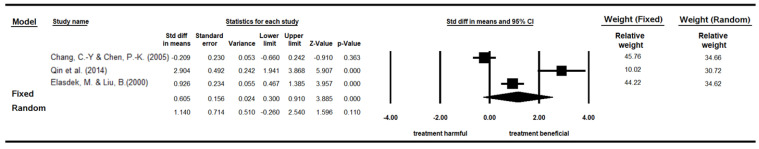
Forest plot of studies on the influence of indoor plants on EEG α waves [56,72,81].

The heterogeneity test of the three studies investigating the influence of indoor plants on EEG α waves revealed a significant difference (*p* < 0.05), with I^2^ = 94.488%, confirming high heterogeneity among the studies. A random-effect model was therefore adopted. The pooled effect size (MD) was 1.140, and the 95% confidence interval ranged from −0.260 to 2.540. The results indicated that the group with plants had greater EEG α waves than the group without plants, but the difference was nonsignificant (*p* = 0.110) (Table 11). The relative weight of both the Chang and Chen [72] and Elasdek and Liu [56] studies was about 34.6%, and that of Qin et al. [81] was 30.72% (Figure 3).

### 3.8. EEG β Waves

Only two papers examining the influence of indoor plants on EEG β waves, which was measured in Hertz by brain activity instruments, were included in this meta-analysis (Table 12). In total, 110 participants were evenly assigned to groups either with plants or without plants. Chang and Chen [72] recruited college students in Taiwan and Qin et al. [81] recruited college students in China. Chang and Chen [72] did not randomly assign their participants to different groups, while Qin et al. [81] did. Both papers were appraised as having moderate research quality.

**Figure 4:**
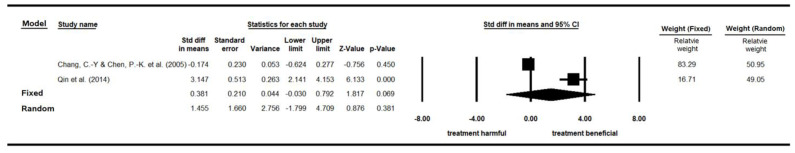
Forest plot of studies on the influence of indoor plants on EEG β waves [72,81].

The heterogeneity test of the two studies investigating the influence of indoor plants on EEG β waves revealed a significant difference (*p* < 0.05), with I^2^ = 97.133%, confirming high heterogeneity between the studies. A random-effect model was therefore adopted. The pooled effect size (MD) was 1.455, and the 95% confidence interval ranged from −1.799 to 4.709. Though the results indicated that the group with plants had greater EEG β waves than the group without plants, the difference was not significant (*p* = 0.381) (Table 13). The relative weight of both the Chang and Chen [72] and Qin et al. [81] studies was about equal, at 50.95% and 49.05%, respectively (Figure 4).

### 3.9. Attention

Three papers examining the influence of indoor plants on attention, which was measured by various cognitive tasks with the unit of measurement as performance scores, were included for the meta-analysis (Table 14). In total, 177 participants were randomly assigned to different groups. Because Larsen et al. [53] divided the participants into two experimental groups (with a high or moderate number of plants) and one control group (without plants), there were 76 participants and 101 participants in the control and experimental groups, respectively. Larsen et al. [53] recruited participants in the United States, Yin et al. [75] recruited adults in the United States, and Shibata and Suzuki [71] recruited college students in Japan. All three papers were appraised as having moderate research quality.

**Figure 5:**
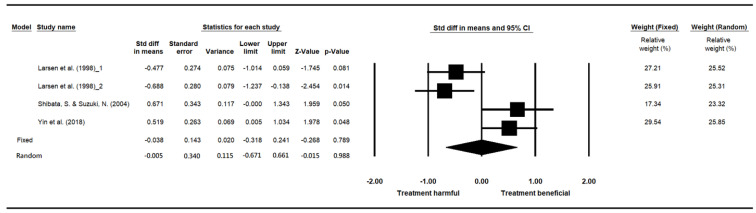
Forest plot of studies on the influence of indoor plants on attention [53,71,75].

The heterogeneity test of the three studies (one with two experimental groups) investigating the influence of indoor plants on attention revealed a significant difference (*p* < 0.05), with I^2^ = 82.088%, confirming high heterogeneity among the studies. A random-effect model was therefore adopted. The pooled effect size (SMD) was −0.005, and the 95% confidence interval ranged from −0.671 to 0.661. The results indicated that the group with plants had lower attention than the group without plants. The difference, however, was not significant (*p* = 0.988) (Table 15). The relative weight of the three studies was relatively similar, ranging from 25.85% to 23.32% (Figure 5).

### 3.10. Academic Achievement

Only two papers examining the influence of indoor plants on academic achievement, which was measured by course grades and examination scores, were included for the meta-analysis (Table 16). The studies had a total of 119 participants. Among these, 58 were in the control group (without plants) and 61 in the experimental group (with plants). Doxey et al. [86] recruited sophomores in the United States, who were not randomly assigned to groups. Han and Hung [66] recruited students from a junior high school in Taiwan, who were randomly assigned to groups. The study of Doxey et al. [86] was appraised as having low research quality, while that of Han and Hung [66] was appraised as having moderate research quality.

**Figure 6:**
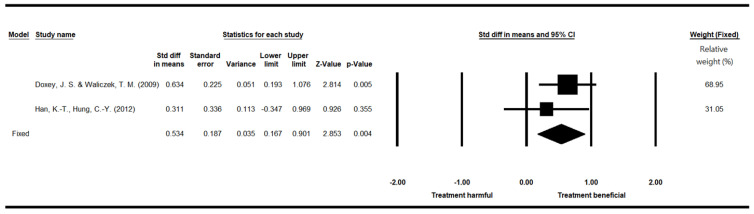
Forest plot of studies on the influence of indoor plants on academic achievement [66,86].

The heterogeneity test of the two studies investigating the influence of indoor plants on academic achievement revealed no significant difference (*p* > 0.05), with I^2^ = 0%, confirming low heterogeneity between the studies. A fixed-effect model was therefore applied. The pooled effect size (SMD) was 0.534, and the 95% confidence interval ranged from 0.167 to 0.901. The results indicated that the group with plants had significantly higher academic achievement (*p* = 0.004) than the group without plants (Table 17). The relative weight of Doxey et al. [86] was 68.95% and that of Han and Hung [66] was 31.05% (Figure 6).

### 3.11. Response Time

Three papers examining the influence of indoor plants on response time, which was measured by various tasks with the unit of measurement as seconds or milliseconds, were included for the meta-analysis (Table 18). These studies had a total of 749 participants. Among them, 374 participants were in the control group (without plants) and 375 in the experimental group (with plants). Nieuwenhuis et al. [58] recruited adult office workers in the United Kingdom, Kim et al. [77] recruited college students in Hong Kong, and Thatcher et al. [94] recruited adults in South Africa. Nieuwenhuis et al. [58] and Thatcher et al. [94] randomly assigned their participants to different groups, while Kim et al. [77] did not. All three papers were appraised as having moderate research quality.

**Figure 7:**
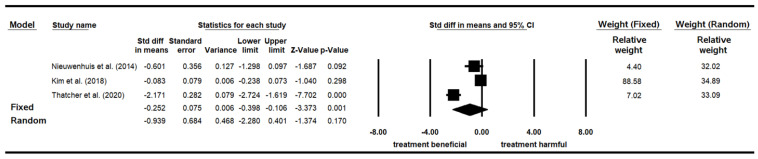
Forest plot of studies on the influence of indoor plants on response time [58,77,94].

The heterogeneity test of the three studies investigating the influence of indoor plants on response time revealed a significant difference (*p* < 0.05), with I^2^ = 96.144%, confirming high heterogeneity among the studies. A random-effect model was therefore adopted. Given that great differences existed between the original data, SMD, rather than MD, was adopted. The pooled effect size (SMD) was −0.939, and the 95% confidence interval ranged from −2.208 to 0.401. The results indicated that the group with plants had less response time than did the group without plants. However, the difference was not significant (*p* = 0.170) (Table 19). The relative weight of the three studies was relatively similar, ranging from 34.89% to 32.02% (Figure 7).

### 3.12. Risk of Bias across Studies

**Figure 8:**
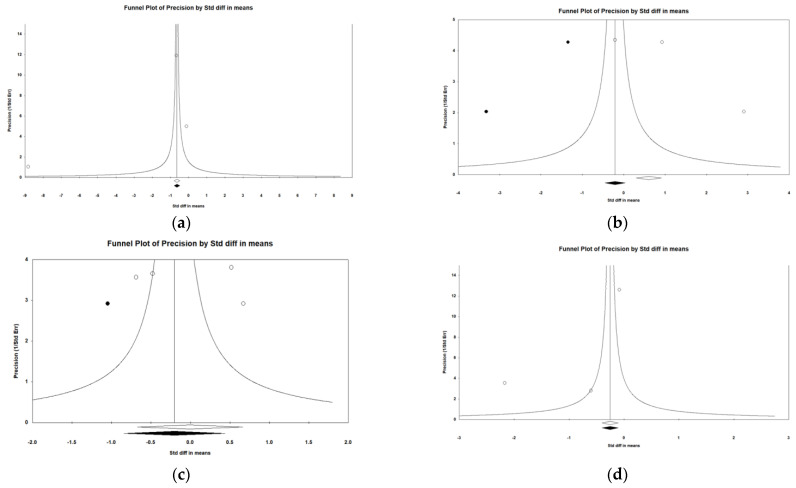
Funnel plots. (**a**) DBP; (**b**) EEG α waves; (**c**) attention; (**d**) response time.

Because at least three records are required for the evaluation of publication bias, only the studies investigating the effects on DBP, EEG α waves, attention, and response time were suitable for testing the risk of bias across records in the meta-analyses. All funnel plots of these studies (Figure 8) revealed a symmetric funnel, confirming the absence of publication bias. Furthermore, the linear Egger’s regressions all indicated no evidence of publication bias (*p* > 0.374) (Table 20).

### 3.13. Additional Analysis

**Figure 9:**
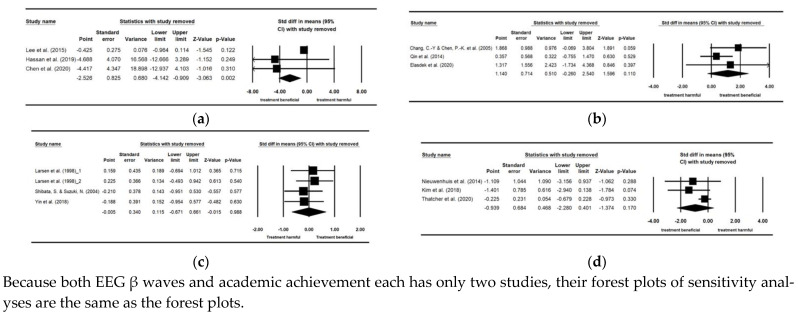
Forest plots of sensitivity analyses. (**a**) DBP [60,76,82]; (**b**) EEG α waves [56,72,81]; (**c**) attention [53,71,75]; (**d**) response time [58,77,94].

Sensitivity analysis was separately performed on records investigating the effects of indoor plants on physiological functions, including DBP and EEG α waves, and those on cognitive functions, including attention and response time, because at least three records are required. None of the pooled effect sizes changed notably when any of the studies was removed (Figure 9). In summary, none of the pooled effect size values in the forest plots exceeded the 95% confidence interval of overall pooled effect size [95]. The results of the aforementioned four meta-analyses were therefore not sensitive; i.e., the results were stable and did not lead to a different conclusion if any of the included studies was deleted.

## 4. Discussion

The 42 records in the present systematic review provide a comprehensive perspective on the topic under investigation. Overall, the review suggests that indoor plants exerted a positive effect on objective functions in participants. Since 90.5% of the records are experiments, the above findings generally support a cause-and-effect relationship [49]. The findings on such matters, such as improved stress-reduction, increased task performance, and improved health, are in accordance with those of the previous reviews [31,36,40,45]. These various reviews together provide converging evidence that indoor plants are beneficial to humans, even though some reviews focused on self-reports, some on objective functions, and some did not distinguish subjective or objective responses. These findings, however, contrast with findings of no improvements in performance and productivity [41] and of no influences of indoor nature on adolescents [35]. This may be because of the differences in the measured outcomes of performance and/or functions and in the ages of the participants (cf. [17]). More studies of the effects of indoor plants on people are needed because only three systematic reviews and one meta-analysis are insufficient to draw conclusive evidence. Moreover, there are some overlapping studies between these reviews, which is not uncommon in reviews. This is also because some studies collected data on both self-reported perceptions and objectively measured responses.

### 4.1. Summary of Evidence

The meta-analyses covered only 16 records, consequently providing a more limited perspective than the systematic review. Nevertheless, it should be noted that synthesis findings are likely to be more reliable than those of single studies. Regarding the physiological functions, the meta-analyses further provided evidence synthesis that (1) participants exposed to indoor plants had significantly lower DBP values, which is related to excitement and arousal [96], than their counterparts exposed to no indoor plants; (2) participants exposed to indoor plants had greater EEG α waves, which is related to relaxation [97], than their counterparts, though the difference was not significant; and (3) participants exposed to indoor plants had greater EEG β waves, which is related to anxiety [69] and attention [98], than their counterparts. This difference was also not significant. It should be noted that whether the EEG wave patterns is a beneficial or an adverse function depends on the context [99]. Regarding cognitive functions, the meta-analyses further provided evidence synthesis that (1) participants exposed to indoor plants had lower attention than their counterparts, though the difference was not significant; (2) participants exposed to indoor plants had significantly higher academic achievement than their counterparts; and (3) participants exposed to indoor plants responded more quickly than their counterparts, though the difference was not significant.

Given that the pooled effect sizes of the records of DBP, EEG α waves, attention, and response time did not change notably when any record was deleted, the meta-analyses had high stability results; i.e., the removal of no study led to a different conclusion. Furthermore, the perfect homogeneity of the two studies on academic achievement provided reliable meta-analysis results (cf. [20]), although one of the included studies had low research quality, which was associated with risks of bias. Some of the results of the meta-analyses, however, were inconsistent, regardless of whether they reached significance. These included greater EEG α and β waves, and lower attention but higher academic achievement and quicker responses when exposed to indoor plants. Since there are three kinds of attention—working memory, cognitive flexibility, and attentional control—researchers should reach a consensus on which task to use to measure attention in order to have a more reliable evidence synthesis [19,20]. More studies of these subjects are needed.

The evidence synthesis of the relaxed physiology, as indicated by significantly lower DBP values when the participants were exposed to indoor plants than their counterparts, provided partial support to the SRT, which proposes that natural environment is helpful for recovery from stress [3], while that of the enhanced cognition, as indicated by significantly higher academic achievement when the participants exposed to indoor plants than their counterparts, provided partial support to the ART, which claims that the natural environment is beneficial to the restoration of directed attention [7]. Although different cultures may influence people’s perceptions of plants and even their functions in relation to plants (cf. [3]), there appears to be no research on these issues. Nevertheless, the evidence synthesis regarding human functions of this study seems not to be influenced by cultures. The evidence synthesis of relaxed physiology comes from the studies recruiting participants in the South Korea [82], China [60], and Taiwan [76]. Although these three studies had a significant heterogeneity (I^2^ = 97.554%), the removal of any study did not change the results. Moreover, the evidence synthesis of enhanced cognition comes from the studies recruiting participants in the US [86] and Taiwan [66] but had a perfect homogeneity (I^2^ = 0%). Nevertheless, more studies are needed to explore the influences of different cultures on peoples’ perceptions and functions with respect to plants.

As mentioned in the previous section concerning the risk of bias within studies, the records suffered, in general, five major risks of bias. First, noncompliance with an ITT analysis might result in unduly liberal estimate of the treatment effect [100]. Second, results obtained from unrepresentative participants might prevent observed effects from being generalizable to a larger population [49], but generalizability is improved more by many heterogeneous small experiments than by only a few large experiments [50]. Third, when outcome assessors were aware of participant allocation, outcomes might be assessed differently [101]. Fourth, statistical power not being reported might increase Type II errors: the acceptance of a null hypothesis that is actually false [102]. Fifth, the lack of appropriate randomization procedures and random allocation to groups might introduce bias [103].

Moreover, the records on the physiological and cognitive functions for the meta-analyses were susceptible to other risks of bias. Inclusion and/or exclusion criteria of participants not being reported [53,58,66,71,77,86,94] might miss the target population and/or might bias the research results [104]. Baseline measures not taken before the intervention [58,72,76,81,86,94] lacked a point of reference to gauge how effective the intervention is [49]. Inappropriate statistical analysis methods for study design, such as repeated-measures or within-subjects design not using repeated-measures or dependent-sample analyses [72,81], led to incorrect results. Not blinding participants to research questions [82] might affect their responses [105]. Lack of individual level allocation [58,81,86], in which each participant did not have an equal opportunity of being assigned to groups, might result in incomparable groups before intervention [50]. Inconsistency of intervention (within and between groups) was an issue in several studies as the intervention included more than one treatment [53,56,58,66,71,72,77,81,94], such as various plant colors.

Some studies found gender differences regarding physiological mobilization [57,62,69,83] and cognitive functions [70,71]. Such findings suggest that taking gender into consideration when investigating the effects of indoor plants is important, since males and females may have differing physiological and psychological responses (cf. [24]). Some of the records also showed different effects of plants with flowers and without flowers on physiology [57,69,83] and behavior [85]. Taking flowers and their colors and even leaf colors into account, therefore, when examining the effects of indoor plants is necessary. Moreover, most of the studies investigated the effect of only single exposure to indoor plants. Although a few studies examined the long-term effects [57,65,66,67,76,86], they did not scrutinize the specific effect of exposure time and/or frequency, nor did they include studies considering the influence of distance between plant and participant.

### 4.2. Limitations

Only journal articles were included in the review and meta-analyses, whereas grey literature was excluded. Therefore, some publication bias may have been involved [43]. There was a chance of positive [86] and/or small [75,81,82] studies being overrepresented, thus biasing the evidence synthesis. In general, studies with negative findings are less published than positive findings [106], which may give a distorted image of what is really known about a subject [107,108]. However, the results of DBP, EEG α waves, attention, and response time all indicated no evidence of publication bias. Furthermore, only a few records were included for the meta-analyses. Though conducting a meta-analysis with two or three studies is acceptable, it is not ideal. Since some of the 42 papers were published a long time ago, their authors could not locate the original data on means and standard deviations. Some authors could not even be reached. Additionally, five of the six meta-analyses had a very high heterogeneity (I^2^ > 82%), which is associated with low reliability results [109]. This may be because of the diversity in the recruited participants, applied interventions, measured outcomes, and adopted study designs (cf. [109]). Additionally, because there were only two or three records for each of the meta-analyses, subgroup analyses, meta-regression analyses, moderating factors (gender, plant quantity, exposure duration, distance to plants, room climate, and room size), and further analyses for the risk of bias could not be conducted. Nevertheless, the results of DBP, EEG α waves, attention, and response time showed no publication bias. Moreover, because of the lack of original data on means and standard deviations or the insufficient number of studies, a meta-analysis on the effects of indoor plants on objective functions in behavior (e.g., pain tolerance and misconduct), health (sick leave, pain killer consumption, and hospitalizations), physiology (EDA, heart rate, respiration rate, and body temperature), and cognition (productivity and reaction) could not be performed. Finally, the studies included for the systematic review and those for the meta-analyses, in general, had moderate research quality (45.3% for those in the review, and 48.0% for those in the meta-analyses; [19]). Thus, high-quality research was lacking (Table 5 and Table 6).

### 4.3. Suggestions

Future studies should recruit more people living in the equatorial area and the Global South in general and Africans in specific, preferably not college students (Table 2). Background information of the participants, such as gender, age, occupation, ethnicity, health status, and number before, during, and after the research, should be provided. Study designs should use more field experiments conducted in real-world indoor environments rather than laboratories in order to improve ecological validity and still maintain sound internal validity [50]. More high-quality research is required, such as research involving experiments that follow the Consolidated Standards of Reporting Trials (CONSORT; [110]), nonrandom experiments that follow the Transparent Reporting of Evaluations with Nonrandomized Designs (TREND; [111]), and, in general, the Publication Manual of the American Psychological Association [112].

Given that indoor plants are the intervention itself, indoor plants are associated with the construct validity of the research [31] in which plant quantity, plant–participant distance, exposure time, and exposure frequency all affect the dose–response relationship (cf. [113,114,115]). Accordingly, we suggest that future studies adopt standardized measurements of the plant quantity, such as the volume percentage of the plants in an indoor environment or the visible greenness rate. The volume percentage of the plants has been used in the included studies [53,67], while the visible greenness rate has also been used in previous studies [38]. The visible greenness rate concerns the percentage of the plants seen by human eyes, which is an objective measurement of plants in a three-dimensional space in the field of vision [116]. Consideration of plant quantity, exposure time, frequency, and distance may assist researchers to examine rigorously how exposure to indoor plants in terms of one event or short-term period or multiple events or long-term periods affects the objective functions of individuals by means of a dose–response or exposure–outcome relationship.

In addition to the dose of and/or exposure to the indoor plants, gender difference, flower colors, flower shapes, plant colors, plant shapes [57,62,69,70,71,83,85,88,89], and cultural influences should also be considered. Furthermore, the physiology of plants, including such factors as their roots and microorganisms [117,118], photosynthesis, adsorption, respiration, and evapotranspiration, which are helpful for air quality and microclimate [40,47,119], may need to be considered. Similarly, researchers should also report more detailed data on room climate, room size, light condition (Table 3 and Table 4), and seasonal condition, because air quality, temperature, relative humidity, light, and season also affect human comfort, performance, and health [120]. The mechanisms of and/or pathways to the effects of indoor plants on human functions also await exploration. Furthermore, indoor plants are mostly studied for their individual performances rather than as a combination. The research into the effect of plants usually focuses on the effects of single plants of different species in different conditions. Attention should further be placed on species that can cohabitate together, thus compensating each other’s needs and recreating the basic forms of symbiosis [121].

Finally, if the number of studies remains inadequate during future analyses, various aspects of human functions may be integrated into physiology (with respect to the sympathetic nervous system or parasympathetic nervous system), cognition (regarding participants’ reaction time and accuracy rate), health (in terms of illness and recovery), and behavior (either positive or negative). In that manner, the standardized mean differences (SMDs) of the function data could be adopted to conduct more rigorous meta-analyses, subgroup analyses, meta-regression analyses, and moderating factors. In contrast to self-reported measures, objective outcome measures lead to fewer reliability and validity concerns (cf. [122]) and risk of bias [123]. Nevertheless, compare and contrast of self-reported measures and objective outcome measures can provide interesting results and can be an advantage of such endeavor (c.f. [124,125,126]).

## 5. Conclusions

The systematic review of 42 records showed that indoor plants affect participants’ objective functions positively, particularly in terms of relaxed physiology and improved cognition. The meta-analyses further provided the evidence synthesis that indoor plants could significantly benefit participants’ SBP and academic achievement, which supported the SRT and ART. The records for the abovementioned meta-analyses, however, were limited, at only three studies for the SBP and two studies for the academic achievement. The evidence synthesis should be interpreted with caution. In brief, the systematic review concluded that, in general, people have better functions with the presence of indoor plants than the absence of indoor plants, and the meta-analyses concluded that, in specific, people have significantly lower SBP and significantly greater academic achievement when indoor plants are present than when indoor plants are not present, though with limited evidence synthesis. Since this study was the first meta-analyses of the effects of indoor plants on people’s functions, however, the findings may help the general public, environmental designers, and planners and policy makers to conduct appropriate assessments and to implement measures to improve psycho-physiological health and productivity (i.e., relaxed physiology and enhanced cognition) of habitants. The estimated productivity decrease caused by sick building syndrome, which is “a medical condition in which people in a building suffer from symptoms of illness or feeling unwell for no apparent reason” [127], in American office workers, for example, was 2%, for an annual cost of roughly 60 billion USD [128]. Furthermore, poor indoor air quality decreases workplace productivity by 10–15% [129]. The integration of plants as a building service is viable. A combination of indoor plants and ventilation technology provides enhanced efficiency and effectiveness of air purification [130,131]. Not only are green spaces needed in cities, but also plants are needed in buildings for people’s health and well-being. For the sake of people’s effective daily functions, indoor plants should be among the important elements of the healthy city, particularly in terms of their easy applicability and accessibility.

## Figures and Tables

**Table 1:** Statistics of published journal articles in Chinese and English during consecutive 5 year periods.

Publication Year	Publication Language				Total	
	Chinese		English			
	Number of Papers	Percentage (%)	Number of Papers	Percentage (%)	Number of Papers	Percentage (%)
1996–2000	1	20	3	8.1	4	9.5
2001–2005	0	0	6	16.2	6	14.3
2006–2010	0	0	7	18.9	7	16.7
2011–2015	1	20	9	24.3	10	23.8
2016–2020	3	60	12	32.4	15	35.7
Total	5	100.0	37	100.0	42	100.0

**Table 2:** Statistics of geographical distribution of the included studies.

Participant Location	Number of Records	Percentage (%)
China (Asia, Global North)	10	23.8
United States (America, Global North)	8	19.0
Japan (Asia, Global North)	6	14.3
South Korea (Asia, Global North)	5	11.9
Taiwan (Asia, Global North)	4	9.5
Norway (Europe, Global North)	3	7.1
United Kingdom (Europe, Global North)	1	2.4
Sweden (Europe, Global North)	1	2.4
Pakistan (Asia, Global North)	1	2.4
Egypt (Africa, Global North)	1	2.4
South Africans (Africa, Global South)	1	2.4
Indonesia (Asia, Equatorial)	1	2.4
Total	42	100.0

**Table 3:** Statistics of experimental conditions.

Experimental Condition		Maximum	Minimum	Number of Records
**Exposure** **Duration**		1 year	15 s.	34
		[76]	[72]	
**Room Size**	Floor area	1260 m^2^	7.26 m^2^	19
		[59]	[77]	
	Volume	675 m^3^	14.52 m^3^	14
		[57]	[77]	
**Distance to Plants**		3 m	0.38 m	13
		[72]	[60]	
**Temperature**		27 °C	20 °C	19
		[78]	[59]	
**Relative Humidity**		70%	34%	13
		[79]	[80]	
**Wind Speed**		0.2 m·s^−1^		1
		[81]		
**Lighting**	Illuminance	1365.5 lux	300 lux	11
		[82]	[56]	
	Quantum	10.6 μmol·m^−2^·s^−1^		1
		[69]		

**Table 5:** Quality appraisal of records in this study.

Quality Indicators		[78]	[53]	[84]	[80]	[83]	[70]	[69]
**Study Design**	Power calculation reported	No	No	No	No	No	No	No
	Inclusion/exclusion criteria reported	No	No	No	No	No	No	No
	Individual level allocation	No	Yes	NA	Yes	Yes	Yes	Yes
	Random allocation to groups/condition/order	**Yes**	Yes	NA	Yes	Yes	Yes	Yes
	Randomization procedure appropriate	**Yes**	Unclear	NA	Unclear	Unclear	Unclear	Unclear
**Confounders**	Groups similar (sociodemographic)	Unclear	Unclear	Unclear	Yes	Yes	Unclear	Yes
	Group balanced at baseline	Unclear	Unclear	Unclear	Unclear	Unclear	Unclear	Yes
	Participants blind to research question	**Yes**	Yes	Unclear	Unclear	Unclear	Unclear	**Yes**
**Intervention Integrity**	Clear description of intervention and control	Yes	Yes	NA	Yes	Yes	Yes	Yes
	Consistency of intervention (within and between groups)	Yes	No	NA	Yes	Yes	Yes	No
**Data Collection Methods**	Outcome assessors blind to group allocation	**No**	**No**	Unclear	Unclear	Unclear	Unclear	**Yes**
	Baseline measures taken before the intervention	Yes	Unclear	NA	Yes	Yes	No	Yes
	Consistency of data collection	Yes	Yes	Yes	Yes	Yes	Yes	Yes
**Analyses**	All outcomes reported (means and SD/SE)	No	Yes	No	No	No	Yes	No
	All participants accounted for (i.e., losses/exclusions)	Yes	Yes	Yes	Yes	Yes	No	Yes
	ITT analysis conducted (all data included after allocation)	Unclear	Unclear	NA	Unclear	Unclear	No	Unclear
	Individual level analysis	Yes	Yes	Yes	Yes	Yes	Yes	Yes
	Statistical analysis methods appropriate for study design	Yes	Yes	Yes	Yes	Yes	Yes	Yes
**External Validity**	Sample representative of target population	No	No	No	No	No	No	No
**Overall Quality Score**	Total number of points (out of possible 38)	20	18	8	20	20	16	22
	Quality rating as percent	52.6 (M)	47.4 (M)	21.1 (L)	52.6 (M)	52.6 (M)	42.1 (M)	57.9 (M)
	Responded to query about “uncertain” ratings	Yes	Yes	NA	No	NA		Yes
**Quality Indicators**		**[85]**	**[71]**	**[72]**	**[55]**	**[68]**	**[63]**	**[67]**
**Study Design**	Power calculation reported	No	No	No	No	No	No	No
	Inclusion/exclusion criteria reported	No	No	Yes	No	No	Yes	No
	Individual level allocation	Yes	Yes	Yes	NA	Yes	Yes	No
	Random allocation to groups/condition/order	Yes	Yes	Unclear	NA	Yes	Yes	No
	Randomization procedure appropriate	Unclear	Unclear	Unclear	NA	Unclear	Unclear	NA
**Confounders**	Groups similar (sociodemographic)	Yes	Unclear	Yes	Unclear	Unclear	Unclear	Yes
	Group balanced at baseline	Unclear	Unclear	Yes	Unclear	Unclear	Unclear	Yes
	Participants blind to research question	Unclear	**Yes**	Unclear	Yes	Unclear	Yes	Unclear
**Intervention Integrity**	Clear description of intervention and control	Yes	Yes	Yes	NA	Yes	Yes	Partial
	Consistency of intervention (within and between groups)	No	No	No	NA	Yes	Yes	No
**Data Collection Methods**	Outcome assessors blind to group allocation	Unclear	**No**	Unclear	NA	Unclear	Unclear	Unclear
	Baseline measures taken before the intervention	Yes	Yes	No	NA	Yes	No	Yes
	Consistency of data collection	Yes	Yes	Yes	Yes	Yes	Yes	Yes
**Analyses**	All outcomes reported (means and SD/SE)	No	Yes	Yes	No	Yes	No	No
	All participants accounted for (i.e., losses/exclusions)	Yes	Yes	Yes	No	Yes	Yes	No
	ITT analysis conducted (all data included after allocation)	Unclear	Unclear	Unclear	NA	Unclear	Unclear	No
	Individual level analysis	Yes	Yes	Yes	Yes	Yes	Yes	Yes
	Statistical analysis methods appropriate for study design	Yes	Yes	No	Yes	Yes	Yes	No
**External Validity**	Sample representative of target population	No	No	No	No	No	No	No
**Overall Quality Score**	Total number of points (out of possible 38)	18	20	18	8	20	20	11
	Quality rating as percent	47.4 (M)	52.6 (M)	47.4 (M)	21.1 (L)	52.6 (M)	52.6 (M)	28.9 (L)
	Responded to query about “uncertain” ratings	NA	Yes				No	No
**Quality Indicators**		**[86]**	**[65]**	**[64]**	**[87]**	**[66]**	**[73]**	**[74]**
**Study Design**	Power calculation reported	No	No	No	No	No	No	No
	Inclusion/exclusion criteria reported	No	Yes	Yes	No	No	Yes	Yes
	Individual level allocation	No	No	Yes	Yes	Yes	Yes	Yes
	Random allocation to groups/condition/order	No	No	Yes	Yes	Yes	Yes	Yes
	Randomization procedure appropriate	NA	Unclear	Unclear	Unclear	Unclear	Unclear	Unclear
**Confounders**	Groups similar (sociodemographic)	Partial	Partial	Unclear	Unclear	Yes	Yes	Yes
	Group balanced at baseline	Unclear	Unclear	Unclear	Partial	Unclear	Yes	Yes
	Participants blind to research question	Unclear	Yes	Yes	Yes	Yes	Unclear	Unclear
**Intervention Integrity**	Clear description of intervention and control	Yes	Yes	Yes	Yes	Yes	Yes	Yes
	Consistency of intervention (within and between groups)	Yes	Yes	Yes	Yes	No	No	No
**Data Collection Methods**	Outcome assessors blind to group allocation	Unclear	No	Unclear	Unclear	No	Unclear	Unclear
	Baseline measures taken before the intervention	No	Yes	No	Yes	Yes	Yes	Yes
	Consistency of data collection	Yes	Yes	Yes	Yes	Yes	Yes	Yes
**Analyses**	All outcomes reported (means and SD/SE)	Yes	No	No	No	No	Yes	Yes
	All participants accounted for (i.e., losses/exclusions)	No	Yes	Yes	Yes	Yes	Yes	Yes
	ITT analysis conducted (all data included after allocation)	No	Unclear	Unclear	Unclear	Unclear	Unclear	Unclear
	Individual level analysis	Yes	Yes	Yes	Yes	Yes	Yes	Yes
	Statistical analysis methods appropriate for study design	Yes	Yes	Yes	Yes	Yes	Yes	Yes
**External Validity**	Sample representative of target population	No	No	No	No	No	No	No
**Overall Quality Score**	Total number of points (out of possible 38)	13	19	20	21	20	24	24
	Quality rating as percent	34.2 (M)	50.0 (M)	52.6 (M)	55.3 (M)	52.6 (M)	63.2 (M)	63.2 (M)
	Responded to query about “uncertain” ratings			No			No	No
**Quality Indicators**		**[88]**	**[89]**	**[79]**	**[58]**	**[81]**	**[82]**	**[54]**
**Study Design**	Power calculation reported	No	No	No	Study 3: No	Yes	No	No
	Inclusion/exclusion criteria reported	Yes	Yes	Yes	Study 3: No	Yes	Yes	Yes
	Individual level allocation	Yes	Yes	Yes	Study 3: No	No	Yes	NA
	Random allocation to groups/condition/order	Yes	Yes	Yes	Study 3: Yes	Unclear	Yes	NA
	Randomization procedure appropriate	Unclear	Unclear	Unclear	Study 3: Unclear	Unclear	Unclear	NA
**Confounders**	Groups similar (sociodemographic)	Yes	Yes	Yes	Study 3: Unclear	Yes	Yes	Unclear
	Group balanced at baseline	Yes	Yes	Yes	Study 3: Unclear	Yes	Yes	Unclear
	Participants blind to research question	**No**	Unclear	Unclear	Study 3: **Yes**	Unclear	No	Unclear
**Intervention Integrity**	Clear description of intervention and control	Yes	Yes	Yes	Study 3: Yes	Yes	Yes	NA
	Consistency of intervention (within and between groups)	No	No	No	Study 3: No	No	Yes	NA
**Data Collection Methods**	Outcome assessors blind to group allocation	**No**	Unclear	Unclear	Study 3: **No**	Unclear	Unclear	Unclear
	Baseline measures taken before the intervention	No	No	No	Study 3: No	No	Yes	NA
	Consistency of data collection	Yes	Yes	Yes	Study 3: Yes	Yes	Yes	Yes
**Analyses**	All outcomes reported (means and SD/SE)	No	No	Yes	Study 3: No	No	No	No
	All participants accounted for (i.e., losses/exclusions)	Yes	Yes	Yes	Study 3: Yes	Yes	Yes	No
	ITT analysis conducted (all data included after allocation)	Unclear	Unclear	Unclear	Study 3: Unclear	Unclear	Unclear	NA
	Individual level analysis	Yes	Yes	Yes	Study 3: Yes	Yes	Yes	Yes
	Statistical analysis methods appropriate for study design	Yes	Yes	No	Study 3: Yes	No	Yes	Yes
**External Validity**	Sample representative of target population	No	No	No	Study 3: No	No	No	No
**Overall Quality Score**	Total number of points (out of possible 38)	20	20	20	Study 3: 14	16	24	8
	Quality rating as percent	52.6 (M)	52.6 (M)	52.6 (M)	Study 3: 36.8 (M)	42.1 (M)	63.2 (M)	21.1 (L)
	Responded to query about “uncertain” ratings	Yes	No	No	Yes			
**Quality Indicators**		**[61]**	**[90]**	**[91]**	**[77]**	**[75]**	**[57]**	**[60]**
**Study Design**	Power calculation reported	No	No	No	No	No	No	No
	Inclusion/exclusion criteria reported	Yes	Yes	No	No	Yes	No	Yes
	Individual level allocation	Yes	Yes	Unclear	Yes	Yes	No	Yes
	Random allocation to groups/condition/order	Yes	Yes	Unclear	Unclear	Yes	No	Yes
	Randomization procedure appropriate	Unclear	Yes	Unclear	Unclear	Unclear	NA	Unclear
**Confounders**	Groups similar (sociodemographic)	Unclear	Unclear	Yes	Unclear	Yes	Unclear	Unclear
	Group balanced at baseline	Unclear	Unclear	Yes	Unclear	Yes	Unclear	Unclear
	Participants blind to research question	Unclear	No	Unclear	Unclear	Unclear	Unclear	Unclear
**Intervention Integrity**	Clear description of intervention and control	Yes	Yes	Yes	Yes	Yes	Yes	Yes
	Consistency of intervention (within and between groups)	Yes	No	No	No	Yes	No	Yes
**Data Collection Methods**	Outcome assessors blind to group allocation	Unclear	Unclear	Unclear	Unclear	Unclear	Unclear	Unclear
	Baseline measures taken before the intervention	No	No	No	Yes	Yes	Yes	Partial
	Consistency of data collection	Yes	Yes	Yes	Yes	Yes	No	Yes
**Analyses**	All outcomes reported (means and SD/SE)	No	No	No	No	No	No	No
	All participants accounted for (i.e., losses/exclusions)	Yes	Yes	Yes	Yes	No	No	Yes
	ITT analysis conducted (all data included after allocation)	Unclear	Unclear	Unclear	Unclear	No	Unclear	Unclear
	Individual level analysis	Yes	Yes	Unclear	Yes	Yes	Yes	Yes
	Statistical analysis methods appropriate for study design	No	No	Unclear	Yes	Yes	No	Yes
**External Validity**	Sample representative of target population	No	No	No	No	No	No	No
**Overall Quality Score**	Total number of points (out of possible 38)	16	16	10	14	22	6	19
	Quality rating as percent	42.1 (M)	42.1 (M)	26.3 (L)	36.8 (M)	58.9 (M)	15.8 (L)	50.0 (M)
	Responded to query about “uncertain” ratings							
**Quality Indicators**		**[76]**	**[59]**	**[56]**	**[92]**	**[62]**	**[93]**	**[94]**
**Study Design**	Power calculation reported	No	No	No	No	No	No	Yes
	Inclusion/exclusion criteria reported	Yes	No	Yes	Yes	No	Yes	No
	Individual level allocation	NA	No	Yes	No	Unclear	Unclear	Yes
	Random allocation to groups/condition/order	NA	No	No	Yes	Unclear	Unclear	Yes
	Randomization procedure appropriate	NA	NA	NA	Unclear	Unclear	Unclear	Unclear
**Confounders**	Groups similar (sociodemographic)	Unclear	Yes	Yes	Unclear	Yes	Unclear	Unclear
	Group balanced at baseline	Unclear	Yes	Yes	Unclear	Yes	Unclear	Unclear
	Participants blind to research question	Unclear	Unclear	Unclear	Unclear	Unclear	Unclear	Yes
**Intervention Integrity**	Clear description of intervention and control	Yes	Yes	Yes	Yes	Yes	Yes	Yes
	Consistency of intervention (within and between groups)	Yes	No	No	Yes	No	No	No
**Data Collection Methods**	Outcome assessors blind to group allocation	Unclear	Unclear	Unclear	Unclear	Unclear	Unclear	Unclear
	Baseline measures taken before the intervention	No	Yes	Yes	Yes	Yes	Yes	No
	Consistency of data collection	Yes	No	Yes	Yes	Yes	Yes	Yes
**Analyses**	All outcomes reported (means and SD/SE)	Yes	No	No	Yes	Yes	Yes	Yes
	All participants accounted for (i.e., losses/exclusions)	Yes	Yes	Yes	No	Yes	No	Yes
	ITT analysis conducted (all data included after allocation)	NA	Unclear	Unclear	No	Unclear	No	Unclear
	Individual level analysis	Yes	Yes	Yes	Yes	Yes	Yes	Yes
	Statistical analysis methods appropriate for study design	Yes	No	Yes	Yes	Yes	Yes	Yes
**External Validity**	Sample representative of target population	No	No	No	No	No	No	No
**Overall Quality Score**	Total number of points (out of possible 38)	16	12	20	18	18	14	20
	Quality rating as percent	42.1 (M)	31.6 (L)	52.6 (M)	47.4 (M)	47.4 (M)	36.8 (M)	52.6 (M)
	Responded to query about “uncertain” ratings							

ITT: intention to treatment; Yes = 2; Partial (Pa.) = 1; No = 0; Unclear (Un) = 0; NA = criterion inapplicable to this study design; any changes made after consultation with study authors are highlighted in boldface. Appraisal quality: High (H): 67–100%, Moderate (M): 34–66%, Low (L): 0–33% [19].

**Table 6:** Statistics of quality appraisal of records in this study.

	Yes		Partial		No		Unclear		NA	
	Frequency	(%)	Frequency	(%)	Frequency	(%)	Frequency	(%)	Frequency	(%)
Power Calculation Reported	2	5	0	0	39	95	0	0	0	0
Inclusion/exclusion Criteria Reported	20	49	0	0	21	51	0	0	0	0
Individual Level Allocation	26	63	0	0	8	20	3	7	4	10
Random Allocation to Groups/Condition/Order	25	61	0	0	6	15	6	15	4	10
Randomization Procedure Appropriate	2	5	0	0	0	0	30	73	9	22
Groups Similar (Sociodemographic)	19	46	2	5	0	0	20	49	0	0
Group Balanced at Baseline	15	37	1	2	0	0	25	61	0	0
Participants Blind to Research Question	11	27	0	0	3	7	27	66	0	0
Clear Description of Intervention and Control	37	90	1	2	0	0	0	0	3	7
Consistency of Intervention (within and between groups)	16	39	0	0	22	54	0	0	3	7
Outcome Assessors Blind to Group Allocation	1	2	0	0	6	15	33	80	1	2
Baseline Measures Taken before the Intervention	22	54	1	2	14	34	1	2	3	7
Consistency of Data Collection	39	95	0	0	2	5	0	0	0	0
All Outcomes Reported (Means and SD/SE)	14	34	0	0	27	66	0	0	0	0
All Participants Accounted for (i.e., losses/exclusions)	32	78	0	0	9	22	0	0	0	0
ITT Analysis Conducted (all data included after allocation)	0	0	0	0	6	15	31	76	4	10
Individual Level Analysis	40	100	0	0	0	0	0	0	0	0
Statistical Analysis Methods Appropriate for Study Design	32	78	0	0	8	20	1	2	0	0
Sample Representative of Target Population	0	0	0	0	41	100	0	0	0	0

**Table 7:** Summary of the outcomes of the records.

Source	Outcomes
[78]	When conducting a computer task, participants had a smaller SBP increase with the presence of plants than without plants. After accomplishing the task, the participants also exhibited a faster SBP decrease when plants were present than when plants were absent. Participants’ reaction time was 12% faster when plants were present than when they were absent.
[53]	Participants had the lowest productivity when the office was furnished with 22 potted plants, whereas the highest productivity was observed when no plants were present.
[84]	Participants had a significantly lower search error rate with indoor greening than without indoor greening.
[80]	The percentage of participants putting their hands in ice water for more than 5 min was higher with the presence of plants than without plants.
[83]	Female participants’ decreases in EEG β waves and EDA were significantly faster when red-flowering geraniums were present than when flowerless geraniums were present and when plants were absent.
[70]	Male participants had a lower score in the association task than their female counterparts when plants were absent, whereas female participants had higher scores on the sorting task regardless of the presence or absence of plants.
[69]	Female participants’ EEG β waves and EDA were significantly lower when flower arrangements were present than when flower arrangements were absent.
[85]	Participants’ time of hand immersion in ice water was significantly longer when green-leaf and flowering plants were simultaneously present than when only green-leaf plants or flowering plants were in the room and when plants were not in the room. Participants’ EDA was significantly lower when the plants were in the room than when the plants were not in the room.
[71]	Female participants showed significantly higher scores of the association task than male participants in the three interventions. Female participants had significantly higher scores of the association task when plants were present than when the magazine-rack was present.
[72]	Participants had the greatest effect of EEG β waves when viewing the slide of the office with a nature window view and indoor plants than other slides.
[55]	A weak but significant correlation was observed between the number of potted plants and sick leave days in the workplace.
[68]	The increased humidity of the indoor potted plants improved the vagus-induced sympathovagal balance of the heart of the participant.
[63]	Participants’ frequency of pain killer consumption, SBP, and heart rate were significantly lower when plants were in the room than when plants were not in the room.
[67]	Participants’ frequency of visiting the school infirmary was significantly lower when plants were in the room than when plants were not in the room.
[86]	Participants’ grade point averages wer significantly higher when plants were present than when plants were absent.
[65]	Participants’ sick leave hours and misconduct were significantly less when plants were present than when plants were absent.
[64]	Participants’ frequency of pain killer use and hospitalization days were significantly lower when plants were in the room than when plants were not in the room.
[87]	Participants’ attention improved significantly from the baseline to after the proofreading task was completed when plants were present, whereas no improvement was noted when plants were absent.
[66]	Participants who took care of plants had greater academic achievement than those who did not.
[73]	Red, yellow, and green plants significantly reduced participants’ DBP and fingertip pulse. Red, purple, and yellow plants significantly reduced participants’ fingertip pulse. Changes in fingertip pulse were more significant in male participants than in female participants.
[74]	Except for yellow African daisies, the other flowers significantly reduced participants’ SBP. Pink and white African daisies, pink and white carnations, and pink and white roses significantly reduced participants’ DBP.
[88]	Male participants spent significantly more time looking at white *Hedera helix* L. than at the dark green variety. Female participants had a greater frequency of looking at yellow-green plants than looking at dark green and green-white plants.
[89]	Male participants spent significantly more time looking at green plants than at red-green ones. The number of fixings at red–green plants was greater than at green and white–green plants. Female participants spent significantly more time looking at green and red–green plants and with greater frequency than green–white plants.
[79]	Relative to green plants with white, yellow, pink, and red flowers, green-leaf plants resulted in a greater increase in participants’ relative slow α power, relative fast α power, relative low β power, and relative moderate β power spectra. By contrast, green-leaf plants with yellow flowers increased participants’ relative θ power spectrum.
[58]	Participants spent less time completing the vigilance and information processing tasks when plants were present than when plants were absent.
[81]	Participants had a significantly higher δ waves and significantly lower α and β waves when plants were present than when plants were absent.
[82]	After transplanting plants, participants had a significantly lower DBP than their counterparts did after a computer operation task.
[54]	The indoor nature contact during work was significantly negatively correlated with sick leave days.
[61]	The percentage of patients with stable blood pressure, heart rate, respiration rate, and body temperature was significantly higher in the ward with plants than in the one without plants. These patients also received a significantly lower dose of pain killers and had significantly shorter hospitalization.
[90]	Yellow–green *Hedera helix* L. received more attention than did the plants of other colors.
[91]	Participants had lower heart rate in the room when the plants were present than when the plants were not present.
[77]	Participants had a significantly faster reaction rate when plants were present than when plants were absent.
[75]	In both the actual and virtual environments with plants, participants exhibited greater changes in SBP, DBP, and EDA than in the plantless environment. They also had greater performance in the visual backward digit span task in the plant setting.
[57]	Participants had the least flicker fusion frequency (eye fatigue) when flowering plants were provided than with other plants and controls.
[60]	Participants had significantly lower SBP and a significant increase in the amplitude of high β waves when plants were present than when plants were absent.
[76]	Participants without houseplants had significantly higher SBP and heart rate than those with houseplants.
[59]	Participants had a significantly greater proportion of significantly decreased pulse rate when the plant was present than when the plant was absent.
[56]	Participants had a significant increase in α relative waves in the prefrontal and occipital lobes and in parasympathetic nervous activity when the plant was present than when the plant was absent.
[92]	There were significant differences between the two horticultural activities and between the pretest and the posttest.
[62]	There were significant differences between the experimental and the control groups in heart rate variability (standard deviation of the NN intervals, root mean square of the successive differences, low frequency, high frequency, and low frequency/high frequency). Within the treatment, male participants’ standard deviation of the NN intervals was significantly different between sowing and transplanting seedlings.
[93]	Participants had a significantly lower heart rate after sowing, transplanting seedlings, and potting succulents. Among the four kinds of horticultural activities, sowing yielded the greatest heart rate reduction while herbal flower potting was the worst.
[94]	Participants had significantly fewer errors and faster time of task completion when the plants and pictures were present than when they were absent.

SBP: systolic blood pressure; DBP: diastolic blood pressure; EEG: electroencephalography; EDA: electrodermal activity.

**Table 8:** Original data of the studies examining the influence of indoor plants on DBP.

Study	Study Design	Appraisal Quality	Without Plant			With Plant		
			*n*	Mean	SD	*n*	Mean	SD
[82]	Experiment (RCT)	Moderate	24	71.75	0.78	24	65.26	0.69
[60]	Experiment (RCT)	Moderate	50	68.2	5.77	50	67.3	9.05
[76]	Survey (non-RCT)	Moderate	300	74.20	6.20	300	70.10	6.00

**Table 9:** Heterogeneity test results of studies on the influence of indoor plants on DBP.

Model	Number of Studies	Pooled Effect Size			Heterogeneity			
		Effect Size	Standard Error	*p*-Value	Q-Value	df (Q)	*p*-Value	I-Squared
Fixed	3	−0.644	0.077	<0.001	81.782	2	<0.001	97.554
Random	3	−2.526	0.825	0.002				

**Table 10:** Original data of the studies examining the influence of indoor plants on EEG α waves.

Study	Study Design	Appraisal Quality	Without Plant			With Plant		
			*n*	Mean	SD	*n*	Mean	SD
[72]	Experiment (non-RCT)	Moderate	38	0.130	0.210	38	0.090	0.170
[81]	Experiment (RCT)	Moderate	17	0.043	0.020	17	0.112	0.027
[56]	Field quasi- experiment (non-RCT)	Moderate	30	0.160	0.054	60	0.210	0.054

**Table 11:** Heterogeneity test results of studies on the influence of indoor plants on EEG α waves.

Model	Number of Studies	Pooled Effect Size			Heterogeneity			
		Effect Size	Standard Error	*p*-Value	Q-Value	df (Q)	*p*-Value	I-Squared
Fixed	3	0.605	0.156	<0.001	36.285	2	<0.001	94.488
Random	3	1.140	0.714	0.110				

**Table 12:** Original data of the studies examining the influence of indoor plants on EEG β waves.

Study	Study Design	Appraisal Quality	Without Plant			With Plant		
			*n*	Mean	SD	*n*	Mean	SD
[72]	Experiment (non-RCT)	Moderate	38	0.160	0.240	38	0.120	0.220
[81]	Experiment (RCT)	Moderate	17	0.051	0.046	17	0.214	0.057

**Table 13:** Heterogeneity test results of studies on the influence of indoor plants on EEG β waves.

Model	Number of Studies	Pooled Effect Size			Heterogeneity			
		Effect Size	Standard Error	*p*-Value	Q-Value	df (Q)	*p*-Value	I-Squared
Fixed	2	0.381	0.210	0.069	34.885	1	<0.001	97.133
Random	2	1.455	1.660	0.381				

**Table 14:** Original data of the studies examining the influence of indoor plants on attention.

Study	StudyDesign	Appraisal Quality	Without Plant			With Plant		
			*n*	Mean	SD	*n*	Mean	SD
[53]_1	Experiment (RCT)	Moderate	28	43.55	6.76	27	40.28	6.94
[53]_2	Experiment (RCT)	Moderate	28	43.55	6.76	26	38.24	8.64
[71]	Experiment (RCT)	Moderate	18	64.67	20.08	18	78.77	21.89
[75]	Experiment (RCT)	Moderate	30	4.69	1.18	30	5.29	1.13

**Table 15:** Heterogeneity test results of studies on the influence of indoor plants on attention.

Model	Number of Studies	Pooled Effect Size			Heterogeneity			
		Effect Size	Standard Error	*p*-Value	Q-Value	df (Q)	*p*-Value	I-Squared
Fixed	4	−0.038	0.143	0.789	16.749	3	0.001	82.088
Random	4	−0.005	0.340	0.988				

**Table 16:** Original data of the studies examining the influence of indoor plants on academic achievement.

Study	Study Design	Appraisal Quality	Without Plant			With Plant		
			*n*	Mean	SD	*n*	Mean	SD
[86]	Field quasi-experiment (non-RCT)	Low	39	2.62	0.847	44	3.14	0.795
[66]	Field experiment (RCT)	Moderate	19	0.133	0.009	17	0.154	0.098

**Table 17:** Heterogeneity test results of studies on the influence of indoor plants on academic achievement.

Model	Number of Studies	Pooled Effect Size			Heterogeneity			
		Effect Size	Standard Error	*p*-Value	Q-Value	df (Q)	*p*-Value	I-Squared
Fixed	2	0.534	0.187	0.004	0.639	1	0.424	0.000
Random	2	0.534	0.187	0.004				

**Table 18:** Original data of the studies examining the influence of indoor plants on response time.

Study	Study Design	Appraisal Quality	Without Plant			With Plant		
			*n*	Mean	SD	*n*	Mean	SD
[58]	Field experiment (RCT)	Moderate	17	20.390	5.870	16	17.390	3.850
[77]	Experiment (non-RCT)	Moderate	317	289.900	51.115	319	286.100	40.377
[94]	Experiment (RCT)	Moderate	40	1228.000	258.720	40	738.650	186.180

**Table 19:** Heterogeneity test results of studies on the influence of indoor plants on response time.

Model	Number of Studies	Pooled Effect Size			Heterogeneity			
		Effect Size	Standard Error	*p*-Value	Q-Value	df (Q)	*p*-Value	I-Squared
Fixed	3	−0.252	0.075	0.001	51.872	2	<0.001	96.144
Random	3	−0.939	0.684	0.170				

**Table 20:** Results of linear Egger’s regressions test.

Egger’s Regression Test		
Effect	Intercept	*p*-Value
DBP	−5.892	0.527
EEG α waves	10.005	0.374
attention	7.251	0.656
response time	−5.679	0.424

## Data Availability

Data are available upon reasonable request.

## Supplementary Materials

The following supporting information can be downloaded at: https://www.mdpi.com/article/10.3390/ijerph19127454/s1, Table S1: The full search strings; Table S2: Full-text excluded, with reason for exclusion.
