# Supplementary material for: Effects of Indoor Plants on Human Functions: A Systematic Review with Meta-Analyses
Source: Int J Environ Res Public Health. 2022 Jun 17;19(12):7454. doi: 10.3390/ijerph19127454 (PMC9224521; doi:10.3390/ijerph19127454)
Supplement: Supplementary file 1 [file ijerph-19-07454-s001.zip › ijerph-1744456-supplementary.pdf]

Supplementary Material

Table S1. The full search strings.

Keywords		
indoor, interior, architecture, building	plant, vegetation, greening, greenery, green, greenness	perception, psychology, emotion, physiology, cognition, restoration, behavior, health, performance

1. indoor AND plant AND perception
2. indoor AND plant AND psychology
3. indoor AND plant AND emotion
4. indoor AND plant AND physiology
5. indoor AND plant AND cognition
6. indoor AND plant AND restoration
7. indoor AND plant AND behavior
8. indoor AND plant AND health
9. indoor AND plant AND performance
10. indoor AND vegetation AND perception
11. indoor AND vegetation AND psychology
12. indoor AND vegetation AND emotion
13. indoor AND vegetation AND physiology
14. indoor AND vegetation AND cognition
15. indoor AND vegetation AND restoration
16. indoor AND vegetation AND behavior
17. indoor AND vegetation AND health
18. indoor AND vegetation AND performance
19. indoor AND greening AND perception
20. indoor AND greening AND psychology
21. indoor AND greening AND emotion
22. indoor AND greening AND physiology
23. indoor AND greening AND cognition
24. indoor AND greening AND restoration
25. indoor AND greening AND behavior
26. indoor AND greening AND health
27. indoor AND greening AND performance
28. indoor AND greenery AND perception
29. indoor AND greenery AND psychology
30. indoor AND greenery AND emotion
31. indoor AND greenery AND physiology
32. indoor AND greenery AND cognition
33. indoor AND greenery AND restoration
34. indoor AND greenery AND behavior
35. indoor AND greenery AND health
36. indoor AND greenery AND performance
37. indoor AND green AND perception
38. indoor AND green AND psychology
39. indoor AND green AND emotion
40. indoor AND green AND physiology
41. indoor AND green AND cognition
42. indoor AND green AND restoration
43. indoor AND green AND behavior
44. indoor AND green AND health
45. indoor AND green AND performance
46. indoor AND greenness AND perception
47. indoor AND greenness AND psychology
48. indoor AND greenness AND emotion
49. indoor AND greenness AND physiology

109. architecture AND plant AND perception
110. architecture AND plant AND psychology
111. architecture AND plant AND emotion
112. architecture AND plant AND physiology
113. architecture AND plant AND cognition
114. architecture AND plant AND restoration
115. architecture AND plant AND behavior
116. architecture AND plant AND health
117. architecture AND plant AND performance
118. architecture AND vegetation AND perception
119. architecture AND vegetation AND psychology
120. architecture AND vegetation AND emotion
121. architecture AND vegetation AND physiology
122. architecture AND vegetation AND cognition
123. architecture AND vegetation AND restoration
124. architecture AND vegetation AND behavior
125. architecture AND vegetation AND health
126. architecture AND vegetation AND performance
127. architecture AND greening AND perception
128. architecture AND greening AND psychology
129. architecture AND greening AND emotion
130. architecture AND greening AND physiology
131. architecture AND greening AND cognition
132. architecture AND greening AND restoration
133. architecture AND greening AND behavior
134. architecture AND greening AND health
135. architecture AND greening AND performance
136. architecture AND greenery AND perception
137. architecture AND greenery AND psychology
138. architecture AND greenery AND emotion
139. architecture AND greenery AND physiology
140. architecture AND greenery AND cognition
141. architecture AND greenery AND restoration

50.	indoor AND greenness AND cognition	142.	architecture AND greenery AND behavior
51.	indoor AND greenness AND restoration	143.	architecture AND greenery AND health
52.	indoor AND greenness AND behavior	144.	architecture AND greenery AND performance
53.	indoor AND greenness AND health	145.	architecture AND green AND perception
54.	indoor AND greenness AND performance	146.	architecture AND green AND psychology
55.	interior AND plant AND perception	147.	architecture AND green AND emotion
56.	interior AND plant AND psychology	148.	architecture AND green AND physiology
57.	interior AND plant AND emotion	149.	architecture AND green AND cognition
58.	interior AND plant AND physiology	150.	architecture AND green AND restoration
59.	interior AND plant AND cognition	151.	architecture AND green AND behavior
60.	interior AND plant AND restoration	152.	architecture AND green AND health
61.	interior AND plant AND behavior	153.	architecture AND green AND performance
62.	interior AND plant AND health	154.	architecture AND greenness AND perception
63.	interior AND plant AND performance	155.	architecture AND greenness AND psychology
64.	interior AND vegetation AND perception	156.	architecture AND greenness AND emotion
65.	interior AND vegetation AND psychology	157.	architecture AND greenness AND physiology
66.	interior AND vegetation AND emotion	158.	architecture AND greenness AND cognition
67.	interior AND vegetation AND physiology	159.	architecture AND greenness AND restoration
68.	interior AND vegetation AND cognition	160.	architecture AND greenness AND behavior
69.	interior AND vegetation AND restoration	161.	architecture AND greenness AND health
70.	interior AND vegetation AND behavior	162.	architecture AND greenness AND performance
71.	interior AND vegetation AND health	163.	building AND plant AND perception
72.	interior AND vegetation AND performance	164.	building AND plant AND psychology
73.	interior AND greening AND perception	165.	building AND plant AND emotion
74.	interior AND greening AND psychology	166.	building AND plant AND physiology
75.	interior AND greening AND emotion	167.	building AND plant AND cognition
76.	interior AND greening AND physiology	168.	building AND plant AND restoration
77.	interior AND greening AND cognition	169.	building AND plant AND behavior
78.	interior AND greening AND restoration	170.	building AND plant AND health
79.	interior AND greening AND behavior	171.	building AND plant AND performance
80.	interior AND greening AND health	172.	building AND vegetation AND perception
81.	interior AND greening AND performance	173.	building AND vegetation AND psychology
82.	interior AND greenery AND perception	174.	building AND vegetation AND emotion
83.	interior AND greenery AND psychology	175.	building AND vegetation AND physiology
84.	interior AND greenery AND emotion	176.	building AND vegetation AND cognition
85.	interior AND greenery AND physiology	177.	building AND vegetation AND restoration
86.	interior AND greenery AND cognition	178.	building AND vegetation AND behavior
87.	interior AND greenery AND restoration	179.	building AND vegetation AND health
88.	interior AND greenery AND behavior	180.	building AND vegetation AND performance
89.	interior AND greenery AND health	181.	building AND greening AND perception
90.	interior AND greenery AND performance	182.	building AND greening AND psychology
91.	interior AND green AND perception	183.	building AND greening AND emotion
92.	interior AND green AND psychology	184.	building AND greening AND physiology
93.	interior AND green AND emotion	185.	building AND greening AND cognition
94.	interior AND green AND physiology	186.	building AND greening AND restoration
95.	interior AND green AND cognition	187.	building AND greening AND behavior
96.	interior AND green AND restoration	188.	building AND greening AND health
97.	interior AND green AND behavior	189.	building AND greening AND performance
98.	interior AND green AND health	190.	building AND greenery AND perception
99.	interior AND green AND performance		
100.	interior AND greenness AND perception		
101.	interior AND greenness AND psychology		
102.	interior AND greenness AND emotion		
103.	interior AND greenness AND physiology		
104.	interior AND greenness AND cognition		
105.	interior AND greenness AND restoration		

106. interior AND greenness AND behavior	191. building AND greenery AND psychology
107. interior AND greenness AND health	192. building AND greenery AND emotion
108. interior AND greenness AND performance	193. building AND greenery AND physiology
	194. building AND greenery AND cognition
	195. building AND greenery AND restoration
	196. building AND greenery AND behavior
	197. building AND greenery AND health
	198. building AND greenery AND performance
	199. building AND green AND perception
	200. building AND green AND psychology
	201. building AND green AND emotion
	202. building AND green AND physiology
	203. building AND green AND cognition
	204. building AND green AND restoration
	205. building AND green AND behavior
	206. building AND green AND health
	207. building AND green AND performance
	208. building AND greenness AND perception
	209. building AND greenness AND psychology
	210. building AND greenness AND emotion
	211. building AND greenness AND physiology
	212. building AND greenness AND cognition
	213. building AND greenness AND restoration
	214. building AND greenness AND behavior
	215. building AND greenness AND health
	216. building AND greenness AND performance

Supplementary Material

Table S2. Full-text excluded, with reason for exclusion.

Excluded paper	Reasons
Allen, J. G., MacNaughton, P., Satish, U., Santanam, S., Vallarino, J., Spengler, J. D. (2016). Associations of cognitive function scores with carbon dioxide, ventilation, and volatile organic compound exposures in office workers: a controlled exposure study of green and conventional office environments. *Environmental Health Perspectives*, 124(6), 805-812.	Unrelated to indoor plants
Dijkstra, K., Pieterse, M. E., & Pruyn, A. (2008). Stress-reducing effects of indoor plants in the built healthcare environment: the mediating role of perceived attractiveness. *Preventive Medicine*, 47, 279-283.	Unrelated to human objective functions
Eldridge, B. M., Manzoni, L. R., Graham, C. A., Rodgers, B., Farmer, J. R., & Dodd, A. N. (2020). Getting to the roots of aeroponic indoor farming. *New Phytologist*, 228(4), 1183-1192.	Unrelated to human objective functions
Evensen, K. H., Raanaas, R. K., Hägerhäll, C. M., Johansson, M., &	Unrelated to human

Pat, G. G. (2017). Nature in the office: an environmental assessment study. *Journal of Architectural and Planning Research*, 34(2), 133-146.	objective functions
Han, K.-T. (2018). Influence of passive versus active interaction with indoor plants on the restoration, behaviour and knowledge of students at a junior high school in Taiwan. *Indoor and Built Environment*, 27(6), 818-830.	Unrelated to human objective functions
Han, H. & Hyun, S. S. (2019). Green indoor and outdoor environment as nature-based solution and its role in increasing customer/employee mental health, well-being, and loyalty. *Business Strategy and the Environment*, 28(4), 629-641.	Unrelated to human objective functions
Hawang, T. & Kim, J. T. (2011). Effects of indoor lighting on occupants' visual comfort and eye health in a green building. *Indoor and Built Environment*, 20(1), 71-90.	Unrelated to indoor plants
Hedge, A., Miller, L. & Dorsey, J. A. (2014). Occupant comfort and health in green and conventional university buildings. *Work*, 49(3), 363-372.	Unrelated to indoor plants
Kim, H.-H., Lee, J.-Y., Yang, J.-Y., Kim, K.-J., Lee, Y.-J., Shin, D.-C., Lim, Y.-W. (2011). Evaluation of indoor air quality and health related parameters in office buildings with or without indoor plants. *Journal of the Japanese Society for Horticultural Science*, 80(1), 96-102.	Unrelated to human objective functions
Kim, H.-H., Park, J.-W., Yang, J.-Y., Kim, K.-J., Lee, J.-Y., Shin, D.-C., Lim, Y.-W. (2010). Evaluating the relative health of residents in newly built apartment houses according to the presence of indoor plants. *Journal of the Japanese Society for Horticultural Science*, 79(2), 200-206.	Unrelated to human objective functions
Kim, H.-H., Yeo, Y.-I., & Lee, J.-Y. (2019). Higher attention capacity after improving indoor air quality by indoor plant placement in elementary school classrooms. *The Horticulture Journal Preview*, doi: 10.2503/hortj.UTD-110.	Unrelated to human objective functions
Lee, A. Y., Kim, S. O., & Park, S. A. (2018). Elementary school students' needs and preferences regarding urban agriculture. *HortTechnology*, 28(6), 783-794.	Unrelated to human objective functions
MacNaughton, P., Satish, U., Laurent, J. G. C., Flanigan, S., Vallarino, J., Coull, B., Spengler, J. D., & Allen, J. (2017). The impact of working in a green certified building on cognitive function and health. *Building and Environment*, 114, 178-186.	Unrelated to indoor plants
MacNaughton, P., Spengler, J., Vallarino, J., Santanam, S., Satish, U., & Allen, J. (2016). Environmental perceptions and health before and after relocation to a green building. *Building and Environment*, 104,	Unrelated to indoor plants

138-144.	
Park, S.-H. & Mattson, R. H. (2009). Ornamental indoor plants in hospital rooms enhanced health outcomes of patients recovering from surgery. *Journal of Alternative and Complementary Medicine*, 15(9), 975-980.	Unrelated to human objective functions
Rogerson, M., Gladwell, V. F., Gallagher, D. J., & Barton, J. L. (2016). Influences of green outdoors versus indoors environmental settings on psychological and social outcomes of controlled exercise. *International Journal of Environmental Research and Public Health*, 13(4), 363. doi: 10.3390/ijerph13040363.	Unrelated to indoor plants
Shih, W.-M., Lin, T.-P., Tan, N.-X., & Liu, M.-H. (2017). Long-term perceptions of outdoor thermal environments in an elementary school in a hot-humid climate. *International Journal of Biometeorology*, 61(1), 1657-1666.	Unrelated to indoor plants
Singh, A., Syal, M., Grady, S. C., Korkmaz, S. (2010). Effects of green buildings on employee health and productivity. *American Journal of Public Health*, 100(9), 1665-1668.	Unrelated to indoor plants
Thatcher, A. & Milner, K. (2014). Changes in productivity, psychological wellbeing and physical wellbeing from working in a 'green' building. *Work*, 49(3), 381-93.	Unrelated to indoor plants
Toyoda, M., Yokota, Y., Barnes, M., & Kaneko, M. (2020). Potential of a small indoor plant on the desk for reducing office workers' stress. HortTechnology, 30(1), 55-63.	Unrelated to human objective functions
Tseng, W. S.-W., Ma, Y.-C., Wong, W.-K., Yeh, Y.-T., Wang, W.-I., & Cheng, S.-H. (2020). An indoor gardening planting table game design to improve the cognitive performance of the elderly with mild and moderate dementia. *International Journal of Environmental Research and Public Health*, 17(5), 1483.	Unrelated to human objective functions
